# Single Doses up to 800 mg of E-52862 Do Not Prolong the QTc Interval – A Retrospective Validation by Pharmacokinetic-Pharmacodynamic Modelling of Electrocardiography Data Utilising the Effects of a Meal on QTc to Demonstrate ECG Assay Sensitivity

**DOI:** 10.1371/journal.pone.0136369

**Published:** 2015-08-20

**Authors:** Jörg Täubel, Georg Ferber, Ulrike Lorch, Duolao Wang, Mariano Sust, A. John Camm

**Affiliations:** 1 Richmond Pharmacology Ltd., St George's University of London, Cranmer Terrace, London, United Kingdom; 2 Statistik Georg Ferber GmbH, Cagliostrostrasse, Riehen, Switzerland; 3 Department of Clinical Sciences, Liverpool School of Tropical Medicine, Liverpool, United Kingdom; 4 Department of Clinical Investigation, ESTEVE Research & Development, Av. Mare de Déu de Montserrat, Barcelona, Spain; 5 Cardiovascular and Cell Sciences Research Institute, St George's University of London, Cranmer Terrace, London, United Kingdom; Indiana University, UNITED STATES

## Abstract

**Background:**

E-52862 is a Sigma-1 receptor antagonist (S1RA) currently under investigation as a potential analgesic medicine. We successfully applied a concentration-effect model retrospectively to a four-way crossover Phase I single ascending dose study and utilized the QTc shortening effects of a meal to demonstrate assay sensitivity by establishing the time course effects from baseline in all four periods, independently from any potential drug effects.

**Methods:**

Thirty two healthy male and female subjects were included in four treatment periods to receive single ascending doses of 500 mg, 600 mg or 800 mg of E-52862 or placebo. PK was linear over the dose range investigated and doses up to 600 mg were well tolerated. The baseline electrocardiography (ECG) measurements on Day-1 were time-matched with ECG and pharmacokinetic (PK) samples on Day 1 (dosing day).

**Results:**

In this conventional mean change to time-matched placebo analysis, the largest time-matched difference to placebo QTcI was 1.44 ms (90% CI: -4.04, 6.93 ms) for 500 mg; -0.39 ms (90% CI: -3.91, 3.13 ms) for 600 mg and 1.32 ms (90% CI: -1.89, 4.53 ms) for 800 mg of E-52862, thereby showing the absence of any QTc prolonging effect at the doses tested. In addition concentration-effect models, one based on the placebo corrected change from baseline and one for the change of QTcI from average baseline with time as fixed effect were fitted to the data confirming the results of the time course analysis.

**Conclusion:**

The sensitivity of this study to detect small changes in the QTc interval was confirmed by demonstrating a shortening of QTcF of -8.1 (90% CI: -10.4, -5.9) one hour and -7.2 (90% CI: -9.4, -5.0) three hours after a standardised meal.

**Trial Registration:**

EU Clinical Trials Register EudraCT 2010 020343 13

## Introduction

Clinical assessments of the QTc interval have become widely utilized in drug investigation [1]; the standards of these required assessments being set out in the ICH E14 guideline and subsequent Q&A documents [2, 3]. The method has been proven to be sensitive in detecting a drug’s potential to cause fatal arrhythmias, yet lacks specificity [4]. TQT studies denote significant cost to the pharmaceutical industry. Therefore valid assessments have been comprehensively discussed to integrate alternative methods in clinical trials [5, 6].

In recent years, amendments or revisions of the S7B and E14 ICH guidelines were under discussion and current proposals [7] include a comprehensive in vitro pro-arrhythmia assay (CiPA) in combination with high precision ECG assessments in Phase I studies. Consequently, Intensive QT trials (IQT) have been developed, which resemble the TQT study designs in many aspects. These trials include triplicate ECGs and multiple collection time points, but they differ from a TQT trial in the smaller number of subjects, the omission of a positive control and at times the lack of a placebo arm.

Literature evidence has suggested that understanding the relationship between the plasma drug concentration and the QT interval can provide important information [8, 9]. In 1976, the first study of pharmacokinetic pharmacodynamic (PK-PD) modelling of drug effects on the QT interval was published by Galeazzi and co-workers where the effects of procainamide on the QT interval were reported [10]. Concentration-effect analysis may have particular value during early phase multiple ascending studies if high-quality ECGs and correlative PK testing can be regularly obtained. The time course analysis as described in ICH E14 has been increasingly supplemented by more sensitive PK-PD analyses [11–14]. Its application requires linearity between the PK-PD relationship concerning plasma concentrations of the drug and its effect on the QT interval without showing hysteresis as prerequisites.

In TQT studies, concentration-response modelling was initially based on the placebo-corrected change from baseline (double difference) [8], and it has recently been extended to parallel group designs [15] allowing for a placebo-corrected prediction of the drug effect at a given plasma concentration with an unbiased standard error. The estimate of a time effect can be used to show ECG assay sensitivity [6, 16, 17]. This second point also makes the use of a model with time effect attractive for crossover studies [6].

Here we describe the application of a concentration-effect analysis validated by meal effects on the ECG to a four-way crossover Phase I study in order to investigate the PK, PD and safety of escalating single doses of E-52862. This compound is a highly selective sigma1 receptor antagonist (S1RA) displaying analgesic activity after systemic administration in preclinical models of pain [18].

## Methods

The protocol for this trial and supporting CONSORT checklist are available as supporting information; see S1 CONSORT Checklist and S1 Protocol.

### Ethics statement

This study (EudraCT: 2010-020343-13) was approved by a National Health Service (NHS) Research Ethics Committee (the Yorkshire Independent Research Ethics Committee) and the Medicines and Healthcare products Regulatory Authority (MHRA), and was conducted in accordance with Good Clinical Practice (GCP) and the Declaration of Helsinki. Each subject received verbal and written information followed by signing off the Informed Consent Form (ICF) prior to any procedures taking place.

### Study Design

The study was conducted at Richmond Pharmacology Ltd located at St George’s University of London between 25 June 2010 and 10 August 2010 and was designed as a double-blind, randomised, placebo-controlled, four-way, crossover study in healthy male and female subjects to evaluate the pharmacokinetics, pharmacodynamics and safety and tolerability of single ascending 500 mg, 600 mg and 800 mg doses of E-52862. General eligibility of 88 subjects for participation in this study was assessed at screening which took place within 14 days of the first study drug administration. Subjects were included if they were male or female, Caucasian, 18–35 years (inclusive) of age, with a body mass index of 18–25 kg/m^2^ (inclusive), using an effective contraceptive method (or were abstinent), judged to be healthy from a medical history, physical examination, routine laboratory investigations and screening ECG assessments. All subjects included in the study had to meet the ECG screening baseline selection criteria and had to be signed off for inclusion by a cardiologist. Thirty two (32) eligible subjects participating in the study attended for screening, four treatment periods (Periods 1–4) and a follow-up visit scheduled 7-14 days after Day 1 of Period 4 (Table 1); subjects were admitted on Day -2 of Period 1 and remained hospitalised until Day 5. No formal size calculation has been performed. Literature data [19] suggests that thorough ECG studies with a sample size of 28 subjects and a SD of 8 ms are capable of detecting 8 ms increase in a QTc (α = 0.05; β = 0.9).

**Table 1:** Summary of study design. Each row corresponds to different treatment sequence providing the balance for period and preceding treatment. E-52862 or Matching Placebo Capsule was taken as a single oral dose in a sitting position with 240 ml of water.

**Period 1**		Washout*	**Period 2**		Washout*	**Period 3**		Washout*	**Period 4**	
**Day -1**	**Day 1**		**Day -1**	**Day 1**		**Day -1**	**Day 1**		**Day -1**	**Day 1**
Placebo	Placebo		Placebo	500 mg		Placebo	600 mg		Placebo	800 mg
Placebo	500 mg		Placebo	Placebo		Placebo	600 mg		Placebo	800 mg
Placebo	500 mg		Placebo	600 mg		Placebo	Placebo		Placebo	800 mg
Placebo	500 mg		Placebo	600 mg		Placebo	800 mg		Placebo	Placebo

*Washout period lasted for 7 days between doses (168 h).

All subjects received four treatments in a randomised four-period crossover design: escalating single doses of 500 mg, 600 mg or 800 mg of E-52862 or placebo. Each period consisted of a placebo baseline ECG day (Day -1) and the treatment day (Day 1) (Table 1). There was at least a seven day washout interval between study drug administrations in Periods 1-4. The ECG measurements on Day -1 were taken at the corresponding clock time points as the ECG measurements and samples for PK analysis on Day 1. The addition of a baseline day enhanced the ECG assessments resulting in a study design which is very similar to a standard single dose crossover TQT study.

Since each subject received the 4 treatments, comparisons between treatment effects were made intra-individually reducing the anticipated variability of the time course analysis. On Days -1 and 1 standardised meals with similar nutritional value were served as follows: lunch (5 h post-dose), dinner (9 h post-dose). The average daily intake was of approximately 2000 kcal with an approximate carbohydrate:protein:fat:fiber ratio of 60:20:10:10% [16, 17]. The composition of these meals was not specifically designed to elicit an ECG response. The meals were identical in each period but not similar between Day-1 and Day 1.

### ECG Assessment and QTc Evaluation

Twelve-lead ECGs were recorded using a MAC1200 (500 samples/second, 4.88 μV amplitude resolution, GE Healthcare, Milwaukee, WI, USA) recorder connected via a fixed network connection to the MUSE Cardiology Information System (MUSE). All ECGs recorded during the study were stored electronically on the MUSE information system. Only ECGs recorded electronically at a stable heart rate were valid for QT interval measurements.

ECG recordings were made at the following time points, pre-dose, 0.25, 0.5, 0.75, 1.0, 1.25, 1.5, 1.75, 2.0, 3.0, 4.0, 5.0, 6.0, 8.0, 12, 24, 48, 72 and 96 h post-dose of each Period (1-4) after the subjects had been resting in a supine position for at least 10 minutes. Clinical staff ensured that subjects were awake during all ECG recordings to avoid autonomic QTc changes occurring during sleep. A semi-permanent skin marker was used to ensure consistent placement of the leads for consecutive study days. At each time point, the ECGs were recorded in triplicate, to reduce variance and improve the precision of measurement. The triplicates were performed at approximately one-minute intervals and each ECG recording (trace) lasted 10 seconds.

### ECG Analysis

Each electronic ECG data file contained the raw waveform ECG data as well as the result of the automated interval measurements performed by the Marquette 12SL ECG Analysis Program (MEAP), software which processes the data within and each of the ECG recorders.

All ECGs and their unconfirmed interval measurements were subsequently reviewed by qualified cardiologists following one of the methods listed in the ICH E14 Guidance for Industry document [2] and ICH E14 Implementation Working Group Questions and Answers document [3] before any of the ECGs were used for the subsequent statistical analyses. This manual adjudication process applied in this study is also referred to in the ICH guidance and relevant literature as “manual over-read”, “computer-assisted”, or “semi-automated” ECG measurements.

The QT interval, RR interval and heart rate, PR interval and QRS duration, the presence or absence of U-waves, quantitative and qualitative ECG variations were assessed by cardiologists with extensive experience with manual on-screen over-reading with electronic callipers using the commercially available MUSE in its latest version to correct any implausible readings presented by the automated process. For all study ECGs (Periods 1-4), the over-reading cardiologists were blinded to time, date, treatment and any data identifying the subject. All ECGs pertaining to an individual volunteer were over-read by the same cardiologist to ensure consistency across all treatments. If manual adjustments of the automated measurement became necessary, a second cardiologist confirmed the assessment.

QT correction by Fridericia's formula (QTcF [QTcF = QT/RR^0.33^]) was used and an individual correction (QTcI) was derived from 12-lead Holter recordings taken for a 24 h screening to generate the baseline values of all four periods (i.e. the mean of all median values per subject per time point on the baseline Day -1) in addition to Fridericia's formula. For each subject, the best individual correction (QTcI) was estimated using linear and log-log-linear models using least square regression models in the following steps:

1. If a model had the smallest absolute correlation coefficient between QTc and RR, the model was selected as the best model.
2. If correlation coefficients were equal, the model with the smallest absolute slope for the regression of QTc on RR was selected as the best correction method.
3. If slopes were equal, the log-log-linear model was selected as the best model.

### Pharmacokinetic assessments

Timings for pharmacokinetic blood sampling were coincident with ECG assessments time points. Plasma samples for determination of the analytes concentration were analysed by Quotient BioAnalytical Sciences, using a validated liquid chromatography/tandem mass spectrometry (LC/MS/MS) method. The pharmacokinetic parameters C_max_, t_max_, t_½_, AUC_0-t_ and AUC_0-∞_ were determined for E-52862 as well as its five metabolites (M1, M2, M3, M4 and M5). They were derived by non-compartmental analysis of the plasma concentration-time data (C_max_, AUC, and t_½_ values were assumed to be log-normally distributed) using SAS version 9.2.

### Statistical Methods

The primary baseline corrections were calculated using averaged QTc baseline values (the mean of all median readings recorded for each time point on the baseline Day -1). This single value (QTc^baselineAV^) was used to calculate ΔQTc for each study period. The effect of E-52862 and metabolites on QTc was calculated as the placebo-subtracted time-matched difference (ΔΔQTc).

To supplement this analysis and to provide, at the same time, some evidence for assay sensitivity, two types of concentration-effect analyses were defined. In each of them, the concentration of E-52862 or one of its five metabolites was used as an explanatory variable.

The first series of models was based on the difference to time-matched placebo of the change of QTcI from average baseline as dependent variable. A linear mixed effects model was used with period, sequence and sex as fixed effects and concentration of one of the six analytes (E-52862, M1, M2, M3, M4, M5) as covariate. A random intercept per subject was also included, and compound symmetry assumed. These series of models are appropriate for a crossover trial, where the time-matched difference to placebo can be obtained for each subject individually. However, an estimated time course corrected for plasma concentration is not allowed. A second series of models was defined as based on the change from average baseline, but without subtraction of the placebo value. Instead, placebo data were used in the model fit in addition to those obtained under the three doses of E-52862. For placebo, concentration values were set to 0. Since the time course (relative to the average baseline) is present in the change from average baseline data, time can and should be introduced as factor in the model [4]. By doing so, the spontaneous time course can be estimated from all subjects and periods, and the regression coefficients for the plasma concentration ("slopes") are corrected for any time course effect and can therefore be considered equivalent to those obtained from "placebo-corrected" values. Apart from the models described above, models with additional random slopes were fitted. For the first series of models, i.e. those based on the difference to time-matched placebo, a model with fixed intercept set to 0 and random intercepts and slopes included was also used. Within the two series, the models with better Akaike information criterion (AIC) were to be used for predictions.

For each of the three doses, the geometric mean across subjects of the intra-individual observed maximum plasma concentration (C_max_) values of each analyte was computed. In agreement with current practice [8], the two-sided 90% CI for the predicted effect at this concentration taken for the appropriate analyte was calculated. This analysis ignores the variability of the C_max_ estimate and therefore there is a risk that the CI for the predicted value is biased. Therefore it was planned that, should the point estimate of the effect at this C_max_ be > 5 ms, the CI would be recalculated using bootstrap methods that take into account the random nature of the mean C_max_. All analyses described above were also repeated for Fridericia's correction.

A test for assay sensitivity based on the predicted effect of lunch served at 5 h was also specified. Based on the analysis of the change from average baseline, two sided 95% CIs for the differences between the two postprandial time points 6 and 8 h to pre-dose were to be completely below nought and the point estimate for the difference was to be well below -5 ms. In the sense of a Bonferroni correction, 95% CIs were used instead of 90% ones in order to correct for multiplicity introduced by the two time points.

The initial analysis were performed using SAS version 9.2. For the supplemental analysis R version 2.13 [20] and in particular the nlme package [21] were used.

## Results

### Subject Demographics

**Fig 1:**
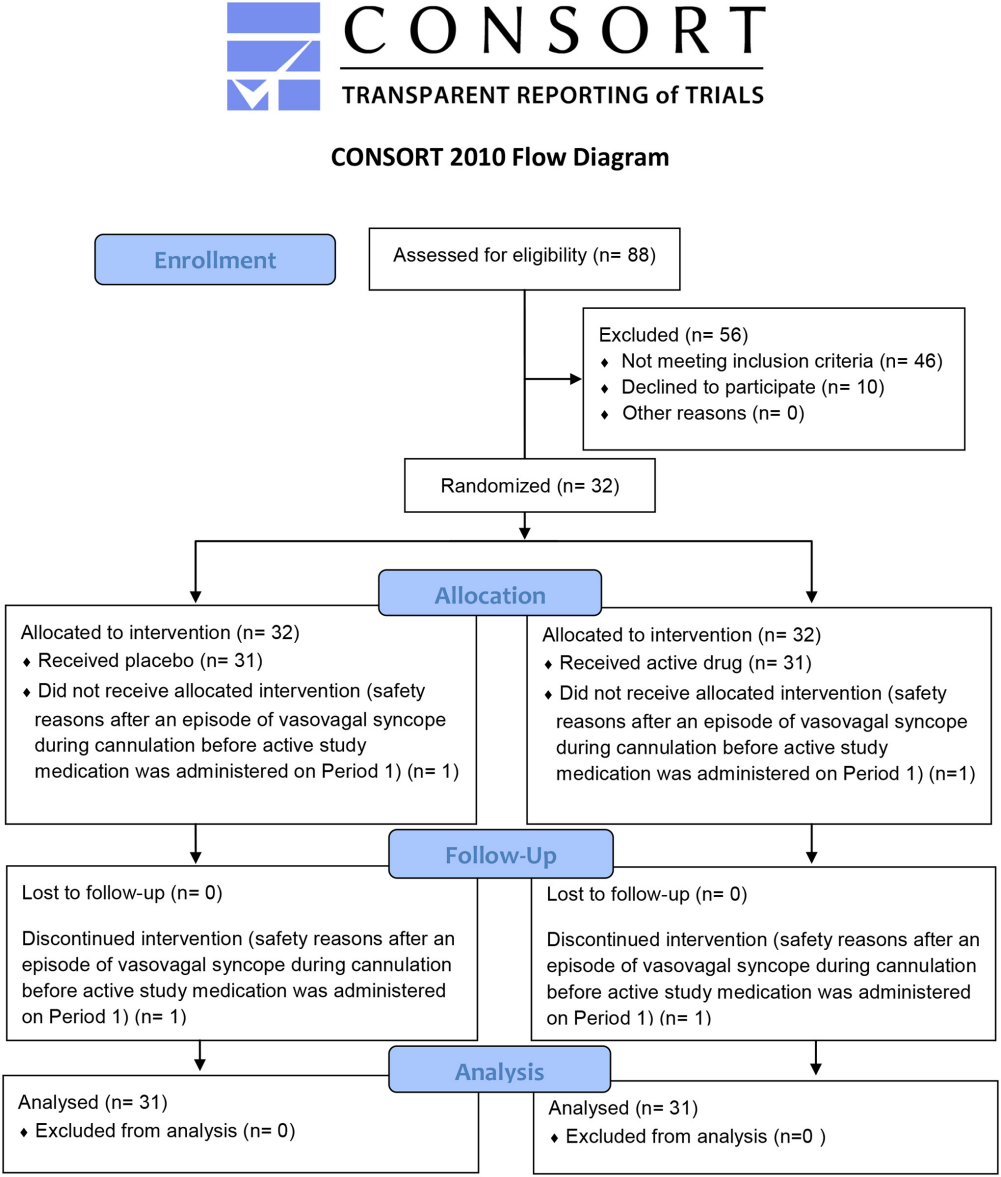
CONSORT 2010 Flow Diagram. Outlined design of the clinical study.

This study was carried out for a period of 7 weeks from the date of first enrolment to the date of last follow-up. The CONSORT 2010 flow diagram is shown in Fig 1. A total of 32 subjects were included in the study and 31 subjects completed all four periods of the study. One subject was withdrawn for safety reasons after an episode of vasovagal syncope during cannulation before administration of active study medication in Period 1. Subject demographics are presented by descriptive statistics in Table 2. Qualitative demographic characteristics (gender, race and ethnicity) are summarised by counts and percentages.

**Table 2:** Subject demographics.

Parameter^a^	Summary Statistics^b^	Overall
**Age (years)**	Mean ± SD	24.22 ± 4.668
**Weight (kg)**	Mean ± SD	67.39 ± 9.654
**Height (cm)**	Mean ± SD	173.09 ± 9.756
**BMI (kg/m** ^**2**^ **)**	Mean ± SD	22.41 ± 1.643
**Female**	Counts (%)	15 (47%)
**Male**	Counts (%)	17 (53%)

^a^All subjects were White and non-Hispanic.

^b^SD (standard deviation).

### Pharmacokinetics

**Fig 2:**
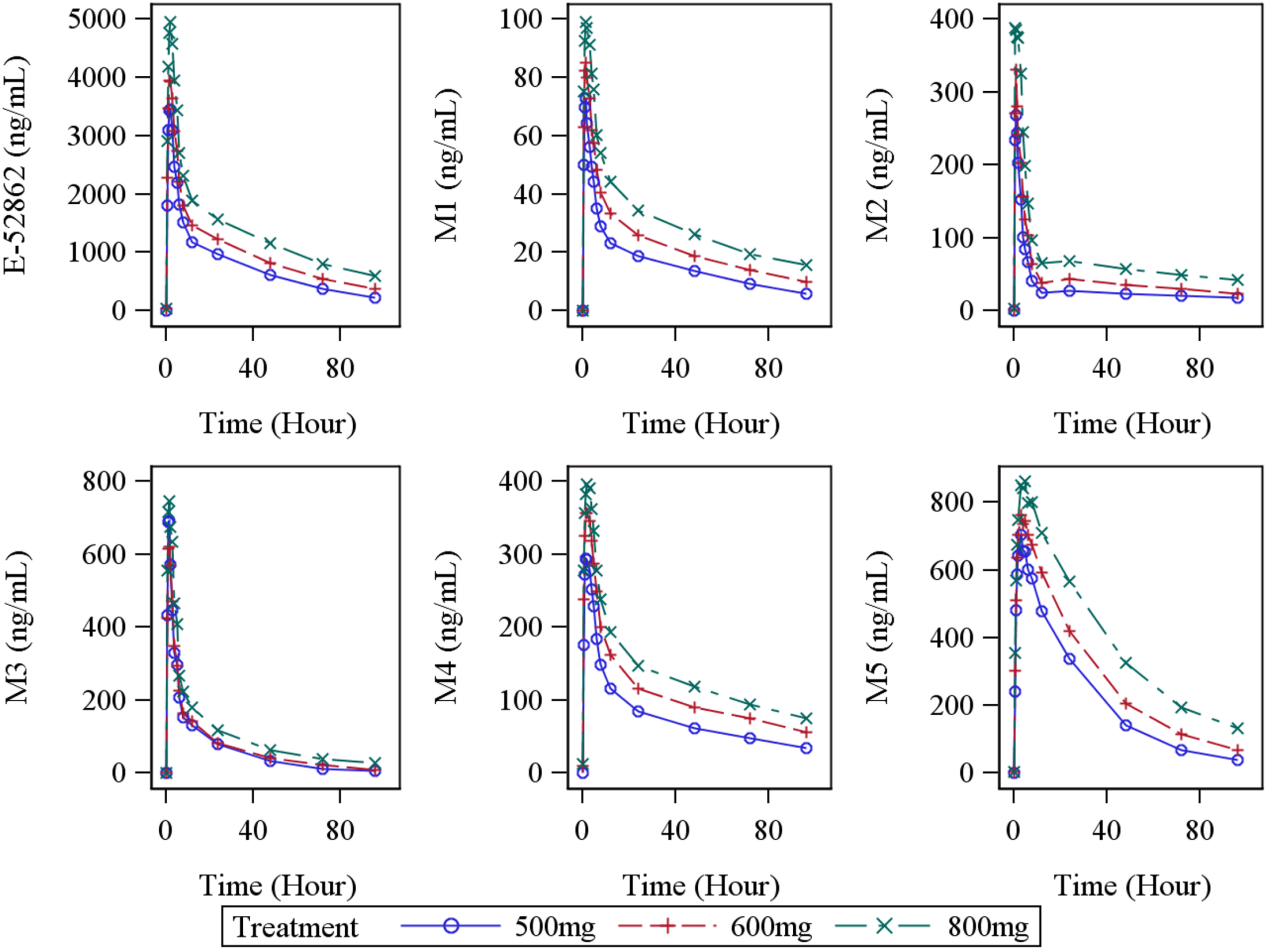
Time course analysis of compound mean concentration. E-52862 and its five metabolites concentration in plasma for three dose levels (500, 600 and 800 mg) is represented.

After single oral dose administration of E-52862 in healthy volunteers, a fast absorption with rapid distribution and slow terminal elimination were observed (Table 3), as published by Abadias et al. [18]. The rate and extent of exposure to E-52862 increased with dose and it was found to be approximately dose proportional for C_max_ and AUC parameters. The five metabolites showed lower (i.e. more than 5 times less) levels of exposure compared to E-52862 in terms of rate and extent, but similar pharmacokinetic profile (Fig 2).

**Table 3:** Pharmacokinetic parameters after single oral dose administration of E-52862.

Dose	C_max_ (ng/mL)	t_max_ (h)^a^	t_1/2_ (h)	AUC_0-t_ (ng.h/mL)	AUC_0-inf_ (ng.h/mL)	AUC_0–24_ (ng.h/mL)	C_max_/Dose (ng/mL)/mg	AUC_0-24_ /Dose (ng.h/mL)/mg
**500 mg**	3938.5 (791.6)	1.50 (0.85)	31.9 (8.0)	75225.0 (14153.6)	85754.7 (19059.2)	36898.5 (6459.7)	7.9 (1.6)	73.8 (12.9)
**600 mg**	4357.9 (709.5)	2.00 (0.61)	35.6 (6.8)	97324.2 (17063.1)	112069.7 (24074.5)	44977.00 (7153.6)	8.7 (1.4)	90.0 (14.3)
**800 mg**	5580.1 (1177.2)	2.00 (0.82)	41.5 (7.1)	130498.5 (20300.9)	155911.0 (28830.9)	56979.7 (8502.9)	11.2 (2.4)	114.0 (17.0)

^a^Mean (SD) for t_max_ values are expressed as median (SD). Time of the last quantifiable concentration (t_last_) was 96 h post-dose.

### Statistical analysis

The models for statistical analysis were defined after completion of the study report. However, a supplemental statistical analysis plan was set up by one of the authors (Georg Ferber) without knowing details of the results or the data of the study and signed off by all parties before releasing the data for analysis.

#### Primary Analysis

The primary ECG analysis of this study compared the mean differences in change from baseline of E-52862 to placebo based changes. The analysis consisted of a per time point analysis of the change from average baseline of QTcI, supplemented by the same analysis using QTcF. This analysis was centred upon the change from average baseline. A linear mixed model with sequence, period, sex, treatment and time and time by treatment interaction as fixed effects, and baseline as covariate was adapted, with subject as random effect, i.e. assuming compound symmetry. Two-sided 90% confidence intervals (CIs) for the difference between results under E-52862 and under placebo were derived at each time point.

All evaluable off-treatment ECGs of each subject (including baseline for Periods 1, 2, 3 and 4) were used for the QT correction analysis. Based on the ‘least square regression model’ QTcI was chosen as the best heart rate correction.

**Fig 3:**
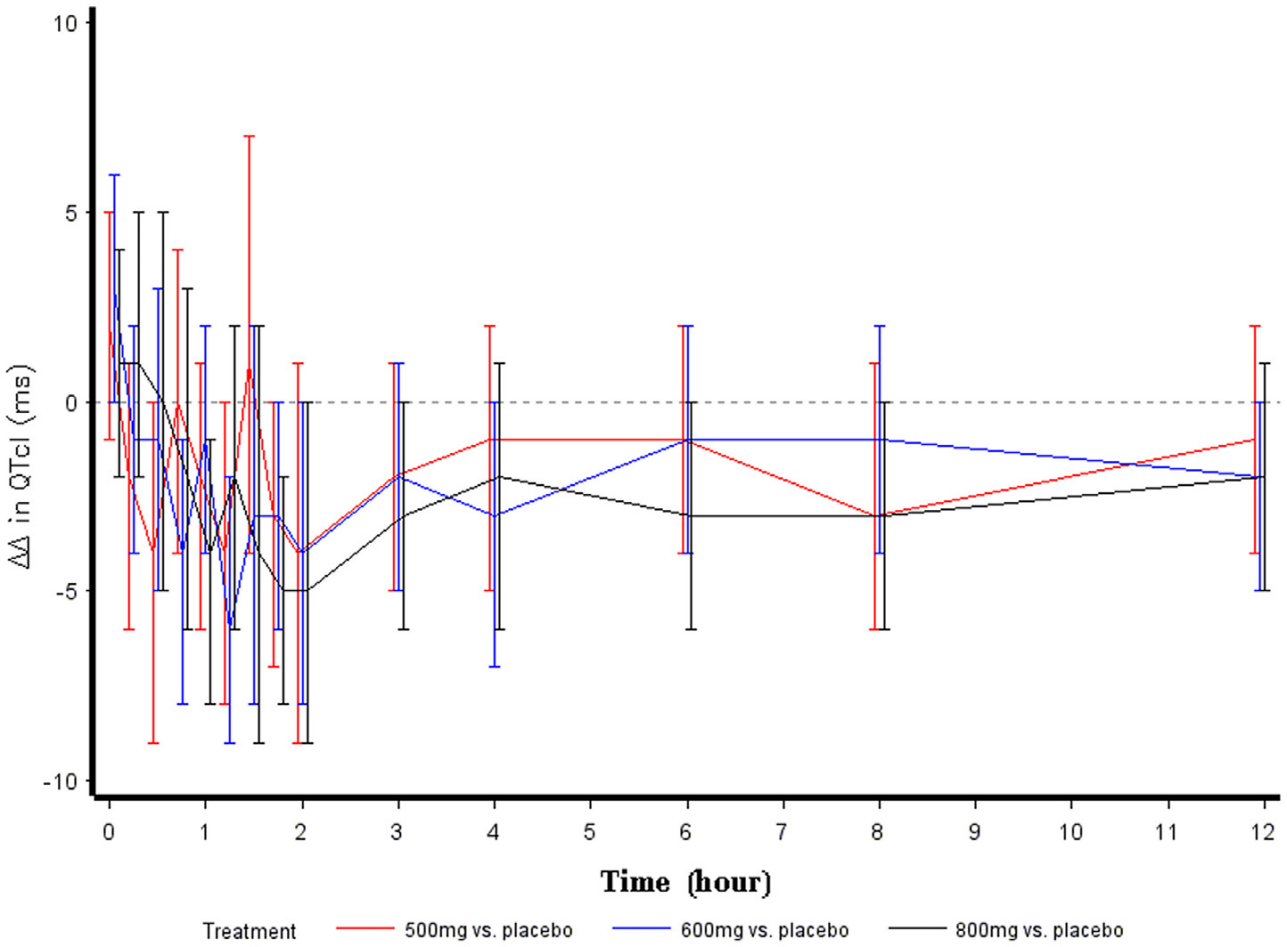
ΔΔQTcI (ms)–primary analysis. Two-sided 90% confidence intervals for mean differences to placebo of change from average baseline.

The QTc analysis comparing the QTcI difference between E-52862 and placebo demonstrated that a single dose of 500 mg, 600 mg and 800 mg E-52862 had no QTc prolonging effect. The largest time-matched QTcI difference between 500 mg E-52862 and placebo was 1.44 ms, between 600 mg E-52862 and placebo was -0.39 ms, and between 800 mg E-52862 and placebo was 1.32 ms. Fig 3 shows the time profile for the mean pair wise differences (ΔΔQTcI) of each E-52862 dose corrected for baseline and placebo QTcI during the first 12 h post-dose period.

#### Models based on time-matched difference to placebo

**Fig 4:**
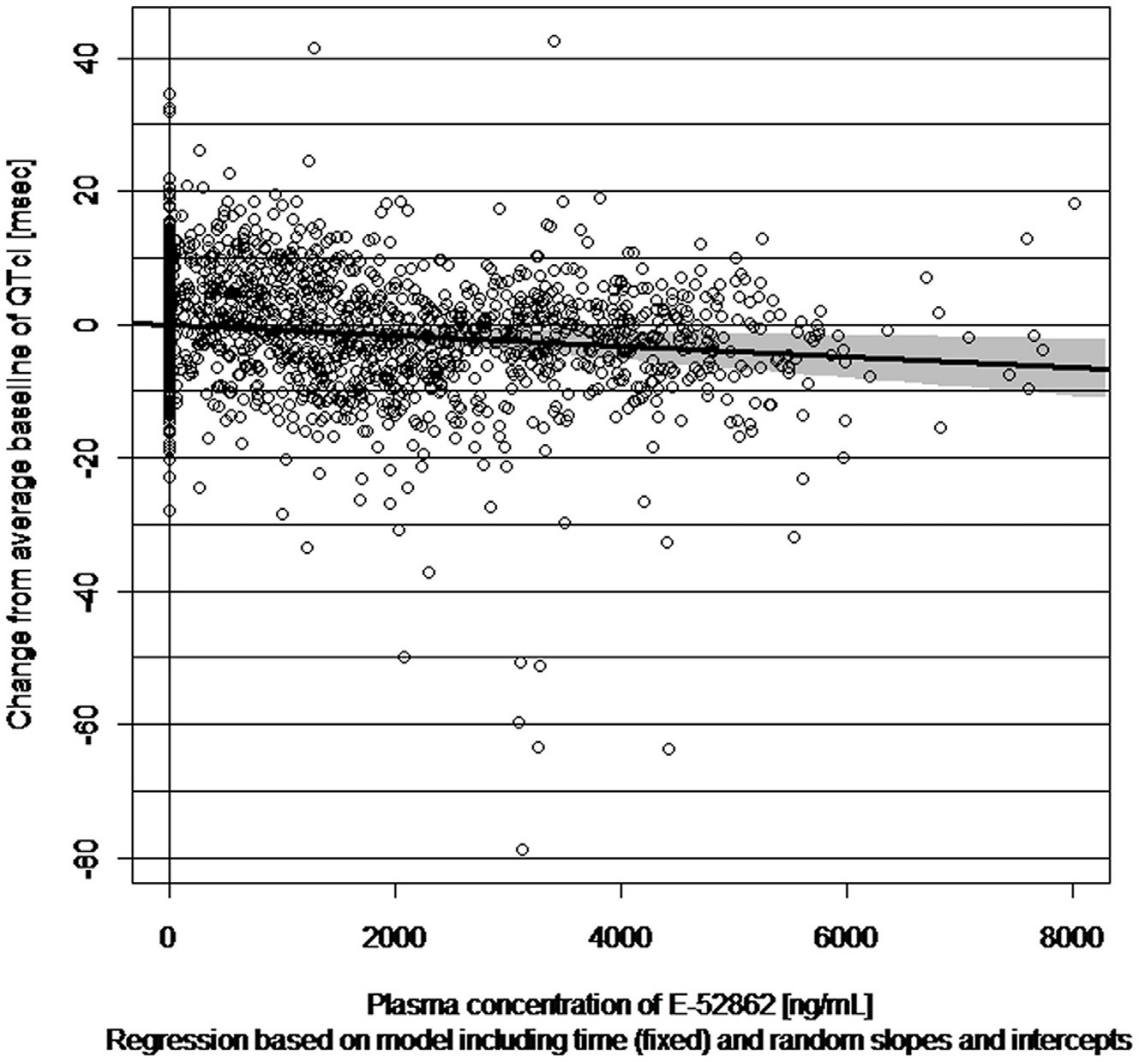
Scatter-plot of difference to time-matched placebo of change from average baseline of QTcI against plasma concentration of E-52862. The regression line and corresponding 90% confidence interval are based on the model with random slopes and intercepts. Most of the outlying values (changes of QTcF from baseline below– 40 ms) come from one subject. If this subject is excluded, the results do not change substantially.

Since all the slopes estimated for QTcI (including all metabolites M1-M5) were negative, particularly in model two, indicating a shortening of QTcI, only the results for the unchanged E-52862 will be presented in detail. Table 4 shows the slope estimates together with a 90% CI for the three models used, both for QTcI and QTcF. It can be observed, that the AIC is nearly the same for all three models, except for model two, i.e. the model with random slope, but allowing for a non-vanishing fixed intercept. This pattern was observed throughout. Likewise, it could be observed that QTcF seemed to give a slightly better fit than QTcI. The results in Fig 4 illustrate the best fit model of QTcI.

**Table 4:** Performance of various concentration-effect models for unchanged E-52862.

QTc Parameter					90% CI for Slope	
	Model^a^	AIC	Best Fit	Slope	lower	upper
**QTcI**	1	8985.8		-0.00032	-0.00068	0.00004
	2	8882.0	*	-0.00024	-0.00101	0.00054
	3	8908.3		-0.00071	-0.00140	-0.00003
**QTcF**	1	8828.7		0.00049	0.00015	0.00083
	2	8768.2	*	0.00051	-0.00011	0.00113
	3	8794.4		0.00009	-0.00041	0.00058

^a^Models based on time matched difference to placebo. Models: 1-period, sequence and sex as factors, random intercept; 2-as above, but including random slope; 3-no fixed effects, random intercept and slope.

* Best Fit

#### Models based on change from average baseline

**Fig 5:**
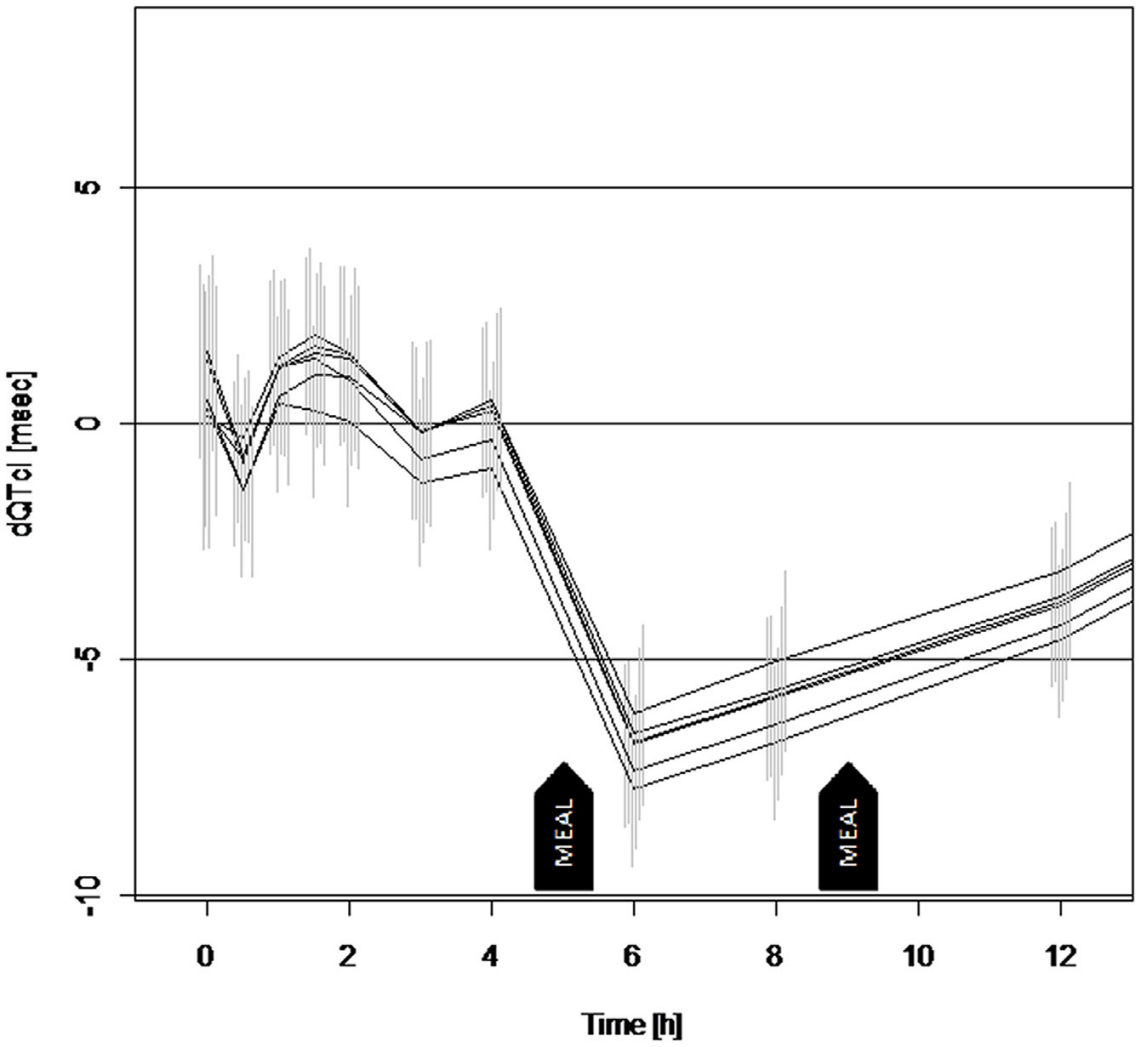
Estimates of the time course of change from average baseline corrected for the concentration of each of the six analytes. The panel represents the estimated time course of the six best fitting models for QTcI during the first 12 h after drug administration. Each line represents the estimates based on the model including random slope for dQTcI and one of the six analytes. The two sided 90% Cl is illustrated for each time point. The models show a good consistency regarding the estimate of time effect. Note the good agreement on the estimators based on the five active moiety and metabolites.

In agreement with the results presented above, the majority of slopes were negative in the second series of models (Table 5). Those for QTcI were significantly negative—apart from the one exception: model two for metabolite M2. A few slopes based on QTcF were positive, but not significant. In all cases, the model with random slopes provided a better fit according to AIC. Table 5 shows the results for unchanged E-52862. The estimated time courses corrected for the plasma concentration of the respective analyte are presented in Fig 5. For the parent and each of the 5 metabolites, the estimate is based on the best fitting model, i.e. the one with random slopes and intercepts.

**Table 5:** Performance of various concentration-effect models for unchanged E-52862. Models based on difference to average baseline and including time points.

QTc Parameter					95% CI	
	Model^1^	AIC	Best Fit	Slope	lower	upper
**QTcI**	1	11661.0		-0.00084	-0.00113	-0.00054
	2	11535.7	*	-0.00079	-0.00143	-0.00016
**QTcF**	1	11299.6		-0.00010	-0.00037	0.00016
	2	11266.3	*	-0.00007	-0.00049	0.00035

^1^ Models: 1-time, period, sequence and sex as factors, random intercept; 2-as above, but including random slope.

* Best Fit

#### Predictions

Only the predictions based on the concentration of unchanged E-52862 are presented in Table 6. Consistently, a QT shortening is predicted, which is somewhat larger if the model based on change from average baseline is used. Since none of the predictions exceeded 5 ms, no bootstrap based confidence intervals were calculated.

**Table 6:** Performance of various concentration-effect models for unchanged E-52862.

Model^a^	QTc Parameter	Dose Group	Concentration (ng/mL)	Prediction (ms)	90% CI	
					lower	upper
**Based on difference to placebo of change from average baseline**	QTcI	500 mg	5464.0	-2.8	-6.3	0.6
	QTcI	600 mg	4302.4	-2.6	-5.2	0.1
	QTcI	800 mg	3859.6	-2.5	-4.8	-0.1
	QTcF	500 mg	5464.0	1.2	-1.2	3.6
	QTcF	600 mg	4302.4	0.6	-1.3	2.4
	QTcF	800 mg	3859.6	0.4	-1.3	2.0
**Based on change from average baseline**	QTcI	500 mg	5464.0	-4.3	-7.2	-1.4
	QTcI	600 mg	4302.4	-3.4	-5.7	-1.1
	QTcI	800 mg	3859.6	-3.1	-5.1	-1.0
	QTcF	800 mg	5464.0	-0.4	-2.3	1.5
	QTcF	600 mg	4302.4	-0.3	-1.8	1.2
	QTcF	500 mg	3859.6	-0.3	-1.6	1.1

^a^Models based prediction of effect on QTc for the observed geometric mean C_max_ of each dose group.

#### Assay Sensitivity

The tests results for assay sensitivity are given in Table 7 showing a shortening of QTcF of -8.1 (90% CI: -10.4, -5.9) 1 h after a meal (6 h post-dose) and -7.2 (90% CI: -9.4, -5.0) 3 h after the meal (8 h post-dose). Since the estimated change from pre-dose at the two time points in the window 2-4 h after the meal are significantly negative, even after correction for multiplicity, they confirm assay sensitivity as defined above.

**Table 7:** Performance of various concentration-effect models for unchanged E-52862. Test of assay sensitivity based on model with time points.

Time point		95% CI		
	QTcF prediction of change from pre-dose (ms)	lower	upper	Significant
**6 h**	-8.1	-10.4	-5.9	*
**8 h**	-7.2	-9.4	-5.0	*

*Significant

## Discussion

### Primary Analyses

The primary ECG analysis compared the mean differences in change from baseline of E-52862 to placebo based changes on QTcI which was chosen as the best correction. The results of the QTc analysis show that a single dose of 500 mg, 600 mg, and 800 mg E-52862 had no QTc prolonging effect. Instead, a small tendency for a shortening of the QTc was observed (Fig 3).

Anticipated plasma concentrations of E-52862 associated with analgesic activity, estimated from preclinical models of pain, suggest that the estimated therapeutic dose range for E-52862 in humans based on C_max_ should be around 100-400 mg and estimated by AUC could be as low as 20-150 mg [18]. Food did not appear to affect the extent of absorption of E-52862, but seemed to delay the absorption rate (unpublished data). The doses administered in this study and the resultant plasma exposures were clearly higher than those expected to be used in efficacy trials and exposures achieved should cover the exposures anticipated in patient trials. In any event, the 800 mg dose showed a much higher incidence of adverse events and it would appear that the range of doses used in this trial would be difficult to exceed in a single dose healthy volunteer trial.

### Linear mixed effects model analyses

The aim of this research was to use a concentration-effect analysis and apply it to a four-way crossover Phase I study to investigate the PK, PD and safety of escalating single doses of a S1RA E-52862 and to use the time course effect of a meal on the ECG as means of testing assay sensitivity. A statistically significant shortening of the QTc was observed at the two available postprandial time points of 6 and 8 h post dose or 1 and 3 h following a meal. This is consistent with our previous work where we have demonstrated that food produces a reliable and consistent QTc shortening effect with a slight variation depending on the heart rate correction used [16] which arises from the postprandial increase in heart rate. The change in QTc was shown to be closely correlated with the release of C-peptide in response to raising blood glucose concentrations after a meal [17] as a result of a physiological response rather than an effect of a blocking drug. In this previous study the concentration-effect analysis has confirmed a QTcF shortening effect with increasing C-peptide concentration [17]. Also concordant with these findings is the fact that correlated with an increase of heart rate, a QTc shortening was observed for 4 h after a first meal by Hnatkova et al. [22]. Further analysis of baseline data showed that, in all 4 periods, the intra-subject variability for 6 and 8 h supports the robustness of this method and consistency of the physiological response triggered by food (S1 File).

The results showed that for both time points, 6 and 8 h, the 95% CIs for the difference to pre-dose were narrow, similar and clearly below 0. These analyses further support the premise that if pre-planned, a carbohydrate rich meal could be a good candidate for assessment of assay sensitivity in a non C-peptide deficient (Type I diabetes) study population.

This study also suggests that linear mixed effects modelling might be particularly useful in early phase dose-escalation studies when assessing the relationship between drug/metabolite plasma concentration and time-matched, baseline-corrected drug-placebo difference in the QT interval. In particular, it should be reinforced that this test for assay sensitivity is based on the same data as the analysis of the drug effect, not on a separate positive control arm. The concentration-effect method used for this study is applicable to any class of medicines, single or multiple doses, irrespective of half-life and large PK variability, including food effects on the PK. Compared to a per time point analysis, this method is more powerful in cases where PK between subjects differs due to variations in absorption or metabolism. Our data also indicate that the fixed algorithm QTcF heart rate correction seems to perform better than QTcI. This might be explained by the additional random variability introduced by the estimation of the individual correction coefficients.

## Conclusions

This study provides further evidence that E-52862 has no QTc prolonging effects. The assay sensitivity of the study was confirmed by using the effects of food on QTc.

The findings from this study show similar results from the well-established per time point analysis and concentration effect modelling methods: absence of any QTc prolongation of regulatory concern and a small tendency for a QTc shortening effect. The power of model based predictions becomes evident when comparing the confidence intervals shown in Fig 3 to those shown in Fig 5. In this crossover study, the concentration analysis based on placebo-corrected values appears to be a better choice due to model simplicity. However, the concentration-effect analysis based on the change from baseline is a powerful alternative to the analysis based on the time-matched difference of placebo. This approach can provide an opportunity for early clinical information on the pro-arrhythmic risk of new drugs as discussed in Ferber et al. [6].

This work further demonstrates that the value of IQT studies is significantly enhanced by the analysis of food effects allowing the benchmarking of the ECG data against a reproducible physiological probe for assay sensitivity [23].

Although ICH guideline E14 does not specify the use of moxifloxacin as a positive control, it has been the most commonly used positive control and the effects on QT interval have been well documented [24]. Currently no method has been accepted to replace this pharmacological positive control. Here we suggest that a standardised meal may be a good alternative for positive control in a situation where a separate arm is not available for the use of moxifloxacin, as in this particular case where a retrospective analysis was conducted, or in a typical Phase I SAD and/or MAD study which will not benefit from a pharmacologic control.

An advantage of the use of food as positive control is the fact that assay sensitivity is tested on the same dataset as the effect of interest. However, more experience from other studies would be desirable to further confirm the appropriateness of this method.

## Supporting Information

S1 CONSORT Checklist Checklist. (DOC)

S1 Protocol Protocol. (PDF)

S1 File QTc changes at 6 and 8h on Day -1. (DOCX)
