# Supplementary material for: Single Doses up to 800 mg of E-52862 Do Not Prolong the QTc Interval – A Retrospective Validation by Pharmacokinetic-Pharmacodynamic Modelling of Electrocardiography Data Utilising the Effects of a Meal on QTc to Demonstrate ECG Assay Sensitivity
Source: PLoS One. 2015 Aug 20;10(8):e0136369. doi: 10.1371/journal.pone.0136369 (PMC4546378; doi:10.1371/journal.pone.0136369)
Supplement: S1 Protocol — (PDF) [file pone.0136369.s002.pdf]

Clinical Study Protocol Amendment (Non-substantial) 2

Sponsor's Reference Number: E-52862

Richmond Pharmacology Study Number: C09079

Esteve Study Code: ESTEVE-SIGM-106

EudraCT Number: 2010-020343-13

**TITLE:**	A double-blind, randomised, placebo-controlled, 4 way cross-over Phase I study to investigate the pharmacokinetics, pharmacodynamics and safety of escalating single doses of E-52862 in young healthy male and female subjects
**SPONSOR:**	Laboratorios del Dr Esteve S.A<br>Av. Mare de Déu de Montserrat, 221 08041<br>Barcelona, Spain<br>Telephone: +34 93 446 6000 Fax: +34 93 456 8774
**PRINCIPAL INVESTIGATOR:**	Dr Jorg Taubel, FFPM, MD<br>Richmond Pharmacology Ltd<br>St George's University of London<br>Cranmer Terrace, Tooting<br>London SW17 0RE, UK<br><br>Richmond Pharmacology Ltd.<br>Mayday University Hospital<br>Thornton Wing, 530, London Road<br>Croydon, CR7 7YE, UK
**Amendment Date**	05 July 2010

Information in this protocol amendment is confidential and should not be disclosed, other than to those directly involved in the execution or the ethical/regulatory review of the study, without written authorisation from Laboratorios del Dr Esteve S.A. (hereinafter known as 'Esteve'), or its affiliates.

Amendment (Non-substantial) 2 Signature Page

Protocol No ESTEVE-SIGM-106:

A double-blind, randomised, placebo-controlled, 4 way cross-over Phase I study to investigate the pharmacokinetics, pharmacodynamics and safety of escalating single doses of E 52862 in young healthy male and female subjects

---

This Clinical Study Protocol and all Amendments have been subjected to an internal Esteve peer review.

I agree to the terms of this study protocol amendment.

Sponsor's Signatory:
Montserrat Abadias, MD

Signature: [signature] **Date:** 7-July-2010

This Clinical Study Protocol and all Amendments have been subjected to an internal Esteve peer review.

I agree to the terms of this protocol and all amendments (Number 2, dated 05 July 2010). I will conduct the study according to the procedures specified herein, and according to the principles of Good Clinical Practice and local regulations.

Principal Investigator:
Dr Jorg Taubel, FFPM, MD

Signature: [signature] **Date:** 07 July 2010

Sponsor:

Laboratorios del Dr Esteve S.A, Av. Mare de Déu de Montserrat, 221 08041, Barcelona, Spain

Centres affected by the Amendment:

Richmond Pharmacology Ltd, UK.

The protocol for the study is to be amended as follows:

Section of protocol affected:

1) Section 8.1, Overall study design and procedures, Table 2, page 20.

2) Section 8.1, Overall study design and procedure, Table 3, page 21.

3) Section 11.1.2, Study Periods 1 to 4, page 33.

4) Section 9.2, Inclusion criteria, page 25.

1) Section 8.1, Overall study design and procedures, Table 2, page 20.

Previous text:

Table 2 Study Plan

**Study Period**	**Screening**	**Period 1-4**							**Follow-Up**
**Study Day**	**-14 to -3**	**-2**	**-1**	**1**	**2**	**3**	**4**	**5**	**7 to 14**
Informed Consent	X								
Demographic Data	X								
Medical History	X								
Physical Examination[a]	X	X							X
Inclusion / Exclusion Criteria[b]	X	X							
Body Weight / Height / BMI[c]	X	X							
Body Temperature	X								X
Blood Pressure / Pulse Rate	X		X	X	X	X	X	X	X
Haematology / Biochemistry	X	X			X			X[d]	X
Serology	X								
Urinalysis	X	X						X[d]	X
Pregnancy Test for Females[e]	X	X							X
Urine Drugs of Abuse Screen[f]	X	X							
Alcohol Breath Test[g]	X	X							
Study Overnight Residence		X	X	X	X	X	X		
IMP Administration			X	X					

12-lead ECG[h]	X	X	X	X	X	X	X	X	
24 Hour 5-lead Holter ECG	X								
Telemetry ECG[i]			X	X					
Cognitive Testing[j]		X[k]	X	X	X				
PK Blood Sampling[h]				X	X	X	X	X	
PK urine collection[l]				X					
Adverse Events Recording	X	X	X	X	X	X	X	X	X
Concomitant Medication Recording	X	X	X	X	X	X	X	X	X

Wash-out between doses in each study period will be at least 7 days (144 hours); Follow-up 7-14 days after the last dose

a A complete physical examination will be performed at screening and follow-up. At admission on Day -2 of Period 1 only, a brief physical examination will be performed.

b Inclusion / Exclusion Criteria will be confirmed at Day -2 of Period 1 only.

c Body weight, height and BMI will be measured at admission on Day -2 of Period 1 only

d Only measured at Day 5 of Period 4.

e Day -2 of Period 1 only.

f Day -2 of Period 1 only.

g Day -2 of Period 1 only.

h Timings are described in Table 3..

i 24-hour transmission and recording after dose on Day -1 and Day1 plus 1 hour pre-dose (25 hours).

j Performed at Days -1 and Day 1 as described in Table 3..

k Training sessions to avoid learning effects will be conducted on Day -2 or Period 1 only.

l PK urine collection from 0-12 hours on Day 1 only in Period 1.

m Meals will be served at standard Unit times as follows:
Day -2: dinner and snack; Day -1 and Day 1: lunch (5 hours post-dose), dinner (9 hours post-dose) and snack (13 hours post-dose)
Day 2 to 5: breakfast (24/48/72/96 hours post dose), lunch (5 hours post-breakfast), dinner (9 hours post breakfast) and snack (11.5 hours post-breakfast).

Revised text:

Table 2 Study Plan

**Study Period**	**Screening**	**Period 1-4**							**Follow-Up**
**Study Day**	**-14 to -3**	**-2**	**-1**	**1**	**2**	**3**	**4**	**5**	**7 to 14**
Informed Consent	X								
Demographic Data	X								
Medical History	X								
Physical Examination[a]	X	X							X
Inclusion / Exclusion Criteria[b]	X	X							
Body Weight / Height / BMI[c]	X	X							
Body Temperature	X								X
Blood Pressure / Pulse Rate	X	**X**[d]	X	X	X	X	X	X	X
Haematology / Biochemistry	X	X			X			X[e]	X
Serology	X								
Urinalysis	X	X						X[e]	X
Pregnancy Test for Females[f]	X	X							X
Urine Drugs of Abuse Screen[g]	X	X							

Alcohol Breath Test[h]	X	X							
Study Overnight Residence		X	X	X	X	X	X		
IMP Administration			X	X					
12-lead ECG[i]	X	X	X	X	X	X	X	X	
24 Hour 5-lead Holter ECG	X								
Telemetry ECG[j]			X	X					
Cognitive Testing[k]		X[l]	X	X	X				
PK Blood Sampling[i]				X	X	X	X	X	
PK urine collection[m]				X					
Adverse Events Recording	X	X	X	X	X	X	X	X	X
Concomitant Medication Recording	X	X	X	X	X	X	X	X	X

Wash-out between doses in each study period will be at least 7 days (144 hours); Follow-up 7-14 days after the last dose

a A complete physical examination will be performed at screening and follow-up. At admission on Day -2 of Period 1 only, a brief physical examination will be performed.

b Inclusion / Exclusion Criteria will be confirmed at Day -2 of Period 1 only.

c Body weight, height and BMI will be measured at admission on Day -2 of Period 1 only

d **Day -2 of Period 1 only.**

e Only measured at Day 5 of Period 4.

f Day -2 of Period 1 only.

g Day -2 of Period 1 only.

h Day -2 of Period 1 only.

i Timings are described in Table 3..

j 24-hour transmission and recording after dose on Day -1 and Day1 plus 1 hour pre-dose (25 hours).

k Performed at Days -1 and Day 1 as described in Table 3.

l Training sessions to avoid learning effects will be conducted on Day -2 or Period 1 only.

m PK urine collection from 0-12 hours on Day 1 only in Period 1.

n Meals will be served at standard Unit times as follows:
Day -2: dinner and snack; Day -1 and Day 1: lunch (5 hours post-dose), dinner (9 hours post-dose) and snack (13 hours post-dose)
Day 2 to 5: breakfast (24/48/72/96 hours post dose), lunch (5 hours post-breakfast), dinner (9 hours post breakfast) and snack (11.5 hours post-breakfast).

2) Section 8.1, Overall study design and procedure, Table 3, page 21.

Previous text:

Table 3 Assessment Time Schedule

Study Day	Protocol Time (hh:mm)	Dose	PK Blood Sampling	ECG	Cognitive Tests	Supine and standing BP/HR
**PERIODS 1 TO 4**						
**-2**				X	X[b]	
**-1**	Predose		X	X		X
	00:00	X				
	00:15			X		

	00:30			X		
	00:45			X		
	01:00			X		X[e]
	01:15			X		
	01:30			X		
	01:45			X		
	02:00			X	X[c]	X
	03:00			X	X[d]	X
	04:00			X		X
	06:00			X		X
	08:00			X		X
	12:00			X		X
1	Pre-dose (24:00)		X	X[a]		X
	00:00	X				
	00:15			X		
	00:30		X	X		
	00:45			X		
	01:00		X	X		X[e]
	01:15			X		
	01:30		X	X		
	01:45			X		
	02:00		X	X	X[c]	X
	03:00		X	X	X[d]	X
	04:00		X	X		X
	05:00		X			
	06:00		X	X		X
	08:00		X	X		X
	12:00		X	X		X
2	24:00		X	X	X	X
3	48:00		X	X		X
4	72:00		X	X		X
5	96:00		X	X		X

a In the statistical analyses, results of pre-dose ECG assessment on Day 1 will also be used for 24 hour baseline ECG assessments, as the two coincide.

b Two training sessions to be performed in Period 1 only.

c To commence once the 2 hour 12-lead ECG recording has finished (to complete 15 minutes before the next) and once the 2 hour vital signs and PK measurements have finished.

d The sustained vigilance test will take place between the 3 and 4 hour post-dose time points.

e At the 1 hour time point, standing blood pressure will not be measured.

Revised text:

Table 3 Assessment Time Schedule

Study Day	Protocol Time (hh:mm)	Dose	PK Blood Sampling	ECG	Cognitive Tests	Supine and standing BP/HR
**PERIODS 1 TO 4**						
**-2**				X	X[b]	X[e]
**-1**	Predose		X	X		X
	00:00	X				
	00:15			X		
	00:30			X		
	00:45			X		
	01:00			X		X[f]
	01:15			X		
	01:30			X		
	01:45			X		
	02:00			X	X[c]	X
	03:00			X	X[d]	X
	04:00			X		X
	06:00			X		X
	08:00			X		X
	12:00			X		X
**1**	Pre-dose (24:00)		X	X[a]		X
	00:00	X				
	00:15			X		
	00:30		X	X		
	00:45			X		
	01:00		X	X		X[f]
	01:15			X		
	01:30		X	X		
	01:45			X		
	02:00		X	X	X[c]	X
	03:00		X	X	X[d]	X
	04:00		X	X		X
	05:00		X			
	06:00		X	X		X
	08:00		X	X		X
	12:00		X	X		X
**2**	24:00		X	X	X	X
**3**	48:00		X	X		X
**4**	72:00		X	X		X

**5**	96:00		X	X		X

a In the statistical analyses, results of pre-dose ECG assessment on Day 1 will also be used for 24 hour baseline ECG assessments, as the two coincide.

b Two training sessions to be performed in Period 1 only.

c To commence once the 2 hour 12-lead ECG recording has finished (to complete 15 minutes before the next) and once the 2 hour vital signs and PK measurements have finished.

d The sustained vigilance test will take place between the 3 and 4 hour post-dose time points.

e Day -2 of Period 1 only.

f At the 1 hour time point, standing blood pressure will not be measured.

3) Section 11.1.2, Study Periods 1 to 4, page 33.

Previous text:

Physical examination, body weight, height and BMI (measured at Period 1 only), 12-lead ECG recording (measured in triplicate), Haematology and Biochemistry, urine drugs of abuse screen and alcohol breath test (Period 1 only), urinalysis, will be performed for all subjects as well as a urine pregnancy test for females (Period 1 only). An AE and concomitant medication check will be performed. Training sessions for the cognitive tests will also be conducted on Day -2 to avoid learning effects.

Revised text:

Physical examination, body weight, height and BMI (measured at Period 1 only), 12-lead ECG recording (measured in triplicate), **Vital signs measurement (Period 1 only),** Haematology and Biochemistry, urine drugs of abuse screen and alcohol breath test (Period 1 only), urinalysis, will be performed for all subjects as well as a urine pregnancy test for females (Period 1 only). An AE and concomitant medication check will be performed. Training sessions for the cognitive tests will also be conducted on Day -2 to avoid learning effects.

4) Section 9.2, Inclusion criteria, page 25.

Previous text:

5 Systolic blood pressure 100–130 mmHg, diastolic blood pressure 60–80 mmHg, and pulse rate 40–90 bpm (all inclusive), measured on the left arm, after 10 minutes in the supine position at screening and at admission on Day -2 of Period 1.

Revised text:

5 Systolic blood pressure ~~100~~**90**–130 mmHg, diastolic blood pressure ~~60~~**50**–80 mmHg, and pulse rate 40–90 bpm (all inclusive), measured on the left arm, after 10 minutes in the supine position at screening and at admission on Day -2 of Period 1.

Reason for Amendment:

1), 2) and 3)

Inclusion of vital signs assessment on Day -2 of Period 1 only in Table 2 and Table 3 which was omitted in error.

4)

The BP range was unnecessarily strict, particularly for female subjects.

Persons who initiated the Amendment:

Richmond Pharmacology Ltd.

CLINICAL STUDY PROTOCOL

Sponsor's Reference Number: E-52862

Richmond Pharmacology Study Number: C09079

Esteve Study Code: ESTEVE-SIGM-106

EudraCT Number: 2010-020343-13

**TITLE:**	A double-blind, randomised, placebo-controlled, 4-way cross-over Phase I study to investigate the pharmacokinetics, pharmacodynamics and safety of escalating single doses of E-52862 in young healthy male and female subjects
**PHASE:**	Phase I
**DRUG:**	E-52862 (as E-52862.HCl)
**SPONSOR:**	Laboratorios del Dr Esteve S.A<br>Av. Mare de Déu de Montserrat, 221 08041<br>Barcelona, Spain<br>Telephone: +34 93 446 6000 Fax: +34 93 456 8774
**PRINCIPAL INVESTIGATOR:**	Dr Jörg Täubel, MD FFPM<br>Richmond Pharmacology Ltd.<br>St George's University of London<br>Cranmer Terrace, Tooting<br>London SW17 0RE, UK<br>Telephone: +44 20 8664 5200 Fax: +44 20 8664 5201
**STUDY SITE:**	Richmond Pharmacology Ltd.<br>St George's University of London<br>Cranmer Terrace, Tooting<br>London, SW17 0RE, UK<br><br>Richmond Pharmacology Ltd.<br>Mayday University Hospital<br>Thornton Wing, 530, London Road<br>Croydon, CR7 7YE, UK
**Protocol Version and Date:**	Final Version (amendment [non-substantial] 2)<br>05 July 2010

Information in this protocol is confidential and should not be disclosed, other than to those directly involved in the execution or the ethical/regulatory review of the study, without written authorisation from Laboratorios del Dr Esteve S.A. (hereinafter known as 'Esteve'), or its affiliates.

1. PROTOCOL APPROVAL SIGNATURES

Final Version (amendment [non-substantial] 2), dated 05 July 2010

Sponsor's Approval

This protocol has been approved by Esteve

Sponsor's Signatory:
Montserrat Abadias, MD

Signature: ______________ **Date:** 12-July-2010

Investigator's Agreement

I have read this Esteve Protocol No. E-52862

A double-blind, randomised, placebo-controlled, 4-way cross-over Phase I study to investigate the pharmacokinetics, pharmacodynamics and safety of escalating single doses of E-52862 in young healthy male and female subjects

I have fully discussed the objectives of this study and the contents of this protocol with Esteve's representative.

I confirm that this study complies with the EMA criteria detailing the guidelines for identifying and mitigating risks for first-in-human clinical trials with investigational medicinal products (EMEA/CMP/SWP/28367/07).

I understand that the information in this protocol is confidential and should not be disclosed, other than to those directly involved in the execution or the ethical/regulatory review of the study, without written authorisation from Esteve. It is, however, permissible to provide information to a subject in order to obtain consent. I agree to conduct this study according to this protocol and to comply with its requirements, subject to ethical and safety considerations and guidelines, and to conduct the study in accordance with ICH guidelines on GCP and with the applicable regulatory requirements.

I understand that Esteve may decide to suspend or prematurely terminate the study at any time for whatever reason; such a decision will be communicated to me in writing. Conversely, should I decide to withdraw from execution of the study I will communicate my intention immediately in writing to Esteve.

Principal Investigator:
Dr Jörg Täubel, MD FFPM

Signature: ______________ **Date:** 12 July 2010

2. STUDY PERSONNEL

Sponsor's Representative	Montserrat Abadias<br>Clinical Research Dept.<br>Laboratorios del Dr. Esteve S.A., Barcelona, Spain<br>Tel: +34 93 446 6000<br>Fax: +34 93 456 8774
Sponsor's Coordinator	Mariano Sust<br>Clinical Research Dept.<br>Laboratorios del Dr. Esteve S.A., Barcelona, Spain<br>Tel: +34 93 446 6000<br>Fax: +34 93 456 8774
Sponsor's Medical Contact	Adelaida Morte<br>Clinical Research Dept.<br>Laboratorios del Dr. Esteve S.A., Barcelona, Spain<br>Tel: +34 93 446 6000<br>Fax: +34 93 456 8774
Principal Investigator	Dr Jörg Täubel, MD FFPM
Co-investigators	Dr Ulrike Lorch, MD FFPM FRCA
Study Site	Richmond Pharmacology Ltd.<br>St George's University of London<br>Cranmer Terrace, Tooting<br>London SW17 0RE, UK<br><br>Richmond Pharmacology Ltd.<br>Mayday University Hospital<br>Thornton Wing, 530, London Road<br>Croydon, CR7 7YE, UK
Clinical Laboratory	The Doctor's Laboratory<br>60 Whitfield Street<br>London W1T 4EU, UK
QP Release	Dr Radivoj Arezina, MD MSc<br>Richmond Pharmacology Ltd.
Bioanalytical Laboratory	Departamento de Bioanálisis y ADME de Desarrollo<br>Laboratorios del Dr. Esteve, S.A.<br>Avda Mare de Deu de Montserrat, 221,<br>08041 Barcelona
Statistics	Dr Duolao Wang, PhD<br>Richmond Pharmacology Ltd.

© ESTEVE (2010) & CONFIDENCIAL

ECG Analysis	Richmond Pharmacology Ltd.
	Professor A. John Camm,<br>Department of Cardiac and Vascular Sciences<br>St George's University of London
Data Management	Dr Radivoj Arezina, MD MSc<br>Richmond Pharmacology Ltd.
Monitoring	Ute Huebner-Otun<br>Metis Solutions Limited<br>16, Golden Ball Lane<br>Maidenhead<br>Berkshire, UK<br>SL6 6NW
Medical Writing	Dr Asif Naseem, PhD<br>Richmond Pharmacology Ltd.

© ESTEVE (2010) & CONFIDENCIAL

3. TABLE OF CONTENTS

1. PROTOCOL APPROVAL SIGNATURES....................2

2. STUDY PERSONNEL....................3

3. TABLE OF CONTENTS....................5

4. LIST OF ABBREVIATIONS....................10

5. STUDY SYNOPSIS....................12

6. INTRODUCTION....................15

6.1 Rationale for conducting study....................15

6.2 Risk-benefit evaluation....................15

7. STUDY OBJECTIVES....................18

7.1 Primary....................18

7.2 Secondary....................18

8. STUDY DESIGN....................18

8.1 Overall study design and procedures....................18

8.1.1 Stopping Criteria....................22

8.2 Rationale for study design, doses and control groups....................23

8.2.1 Choice of Subjects for Study....................23

8.2.2 Dose Regimens of Administration....................23

8.2.3 Justification of Doses....................23

8.2.4 Monitoring and Communication of Adverse Events / Adverse Drug Reactions....................24

8.2.5 Investigator Site Facilities and Personnel....................24

9. SELECTION AND WITHDRAWAL OF SUBJECTS....................24

9.1 Number and Source of Subjects....................24

9.2 Inclusion Criteria....................24

9.3 Exclusion Criteria....................26

9.4 Subject Restrictions....................28

9.5 Subject inclusion and randomisation....................29

9.6 Randomisation....................29

9.7 Withdrawal of Subjects....................29

© ESTEVE (2010) & CONFIDENCIAL

9.7.1 Criteria for withdrawal 29
9.7.2 Procedures for subject withdrawal 29

10. STUDY AND CONCOMITANT TREATMENTS 30

10.1 Investigational Medicinal Products (IMPs)....30
10.1.1 Doses and treatment regimen....30
10.2 Labelling of Investigational Medicinal Products....31
10.3 Drug Accountability....31
10.4 Storage 31
10.5 Blinding and Procedures for Unblinding the Study 31
10.5.1 Methods for ensuring blinding....31
10.5.2 Methods for unblinding the study 31
10.6 Concomitant Medications 31
10.7 Treatment compliance 32

11. STUDY PROCEDURES 32

11.1 Schedule of Study Procedures 32
11.1.1 Screening visit....32
11.1.2 Study Periods 1 to 4....33
11.1.3 Follow-up....35
11.2 Recording of data 35

12. STUDY METHODOLOGY 35

12.1 Laboratory Safety Measurements....36
12.1.1 Haematology, biochemistry....36
12.1.2 Serology 36
12.1.3 Urinalysis....36
12.1.4 Pregnancy test 36
12.1.5 Drugs of abuse....36
12.1.6 Physical examinations....37
12.2 Alcohol Breath Test 38
12.3 Vital Signs....38
12.3.1 Blood pressure and pulse rate 38
12.3.2 Body temperature....38
12.4 ECG Measurements 38
12.4.1 Recording of 12-lead ECG 38
12.4.2 Analysing and over-reading 12-lead ECG....39
12.4.3 Recording of 24 hour 5-lead Holter ECG 39

© ESTEVE (2010) & CONFIDENCIAL

12.4.4 Extracting, Analysing and Over-Reading Holter ECG 40
12.4.5 Telemetry 40
12.5 Cognitive tests 40
12.6 Pharmacokinetic Measurements 40
12.6.1 Collection of pharmacokinetic samples 41
12.6.2 Determination of drug concentrations in pharmacokinetic samples 41
12.7 Volume of Blood Sampling 41
13. ADVERSE EVENTS 42
13.1 Adverse Events 42
13.1.1 Definitions 42
13.1.2 Recording of adverse events 44
13.1.3 Assessment of adverse events 44
13.1.4 Reporting of SAEs 46
14. QUALITY ASSURANCE AND QUALITY CONTROL 47
14.1 Quality Assurance (QA) and Quality Control (QC) 47
14.2 Monitoring 47
15. DATA MANAGEMENT 48
15.1 Case Report Forms 49
16. STATISTICAL EVALUATION AND CALCULATION 49
16.1 Pharmacokinetic Evaluation 49
16.1.1 Calculation or derivation of pharmacokinetic variables 49
16.1.2 Analysis of pharmacokinetic variables 50
16.2 ECG Evaluation 50
16.2.1 Calculation or derivation of ECG parameters 51
16.2.2 Statistical methods for ECG analyses 51
16.3 Cognitive test analysis 52
17. STATISTICAL METHODS AND SAMPLE SIZE DETERMINATION 52
17.1 Statistical Analysis Plan 52
17.2 Analysis Sets 53
17.2.1 General principles 53
17.2.2 Safety analysis set 53
17.2.3 Pharmacokinetic analysis set 53
17.3 Analysis of Other Safety Parameters 53

© ESTEVE (2010) & CONFIDENCIAL

17.3.1 Subject demographics 53
17.3.2 Adverse events 53
17.3.3 Laboratory parameters 54
17.3.4 Vital signs 54
17.4 HANDLING OF MISSING AND INCOMPLETE DATA 54
17.4.1 Pharmacokinetic data 54
17.4.2 ECG data 54
17.4.3 Cognitive test data 54
17.5 Sample size 54

18. SPONSOR'S AND INVESTIGATOR'S RESPONSIBILITIES 54
18.1 Sponsor's Responsibilities 55
18.1.1 GCP compliance 55
18.1.2 Indemnity/liability and insurance 55
18.1.3 Protocol management 55
18.1.4 End of trial notification 55
18.1.5 Submission of summary of clinical trial report to competent authorities of member states concerned and the Independent Ethics Committees 55
18.2 Investigator's Responsibilities 56
18.2.1 GCP compliance 56
18.2.2 Regulatory approval 56
18.2.3 Protocol adherence and Investigator agreement 56
18.2.4 Documentation and retention of records 56
18.3 Ethical Considerations 57
18.3.1 Informed consent 57
18.3.2 Institutional Review Board or Independent Ethics Committee approval 57
18.4 Confidentiality 58
18.5 Publication Policy 58

19. REFERENCES 60

20. APPENDICES 62

List of Tables

Table 1 Study Flow Chart....19
Table 2 Study Plan....20
Table 3 Assessment Time Schedule....21
Table 4 Laboratory Safety Variables that will be Measured....37
Table 5 Volume of Blood to be Drawn from Each Subject....41

4. LIST OF ABBREVIATIONS

Abbreviation	Explanation
σ	Sigma
ABPI	Association of British Pharmaceutical Industry
ADR	Adverse drug reaction
AE	Adverse event
AF	Atrial fibrillation
AUC	Area under the plasma concentration versus time curve
BMI	Body mass index
BP	Blood pressure
Ca^{2+}	Calcium
C_{max}	The observed maximum plasma concentration
CNS	Central Nervous System
CPU	Clinical Pharmacology Unit
CRA	Clinical Research Associate
CRF	Case Report Form
CSR	Clinical Study Report
DCF	Data Clarification Form
DHP	Data handling protocol
ECG	Electrocardiogram
EMA	European Medicines Agency
EU	European Union
GCP	Good Clinical Practice
GMP	Good Manufacturing practice
HIV	Human immunodeficiency virus
IB	Investigator Brochure
ICF	Informed Consent Form
ICH	International Conference on Harmonisation
IEC	Independent Ethics Committee
IMP	Investigational Medicinal Product
IRB	Institutional Review Board
LLOQ	Lower limit of quantification
LOQ	Limit of quantification
MEAP	Marquette® 12SL™ ECG Analysis Program
MedDRA	Medical Dictionary for Regulatory Activities

© ESTEVE (2010) & CONFIDENCIAL

MID	Minimum tolerated dose
MTD	Maximum tolerated dose
NCI	National Cancer Institute
NOAEL	No Observed Adverse Effect Level
OTC	Over-the-counter
PK	Pharmacokinetic
PR	Pulse rate
PR interval	The PR interval is the time from the beginning of the P wave to the beginning of the QRS complex.
QA	Quality Assurance
QC	Quality control
QP	Qualified Person
QRS	Ventricular depolarization interval taken as from the beginning of the Q or R Wave (when Q is absent) to J point
QT	The sum of ventricular depolarization and repolarisation taken as from the start of ventricular depolarization to end of repolarisation
QTc	QT corrected values
QTcB	QT interval corrected for heart rate using Bazett's formula
QTcF	QT interval corrected for heart rate using Fridericia formula
QTcI	QT interval corrected for heart rate using individual correction
RPL	Richmond Pharmacology Ltd
RR	Interval between one point of a QRS complex and the corresponding point of the preceding QRS complex
SAD	Single ascending dose
SAE	Serious adverse event
SAP	Statistical Analysis Plan
SOM	Study Operations Manual
SOP	Standard Operating Procedure
SUSAR	Suspected Unexpected Serious Adverse Reaction
TDL	The Doctors Laboratory
TQT	Thorough QT/QTc
T-wave	The T-wave represents the repolarisation (or recovery) of the ventricles. The interval from the beginning of the QRS complex to the apex of the T wave is referred to as the absolute refractory period.
U-wave	The U-wave is not always seen. It is typically small, and, by definition, follows the T wave. U waves are thought to represent repolarisation of the papillary muscles or Purkinje fibres.

© ESTEVE (2010) & CONFIDENCIAL

5. STUDY SYNOPSIS

**Protocol Ref.** 2010-020343-13	**Study drug:** E-52862

Title of the study:

A double-blind, randomised, placebo-controlled, 4-way cross-over Phase I study to investigate the pharmacokinetics, pharmacodynamics and safety of escalating single doses of E-52862 in young healthy male and female subjects

Principal Investigator:
Dr Jörg Täubel, MD FFPM

Study centre:
Richmond Pharmacology Ltd, St George's University of London, Cranmer Terrace, London, SW17 0RE.

Richmond Pharmacology Ltd, Mayday University Hospital, Thornton Wing, 530, London Rd, Croydon, CR7 7YE.

**Study period (planned):** Summer 2010	**Clinical phase:** 1

Objectives:

Primary

- To assess the safety and tolerability of single ascending 500 mg, 600 mg and 800 mg doses of E-52862

Secondary

- To describe the cardiovascular safety profile, including rhythm and conduction abnormalities, categorical QT/QTc interval data and qualitative and quantitative ECG variations from baseline
- To describe and compare the number and the rates of adverse events under each treatment
- To describe the pharmacokinetic profiles (PK) of E-52862 and metabolites in the study population
- To describe dose response relationship, using a battery of cognitive tests

Study Design:

This will be a single centre, double blind, randomised, placebo-controlled, four-way, cross-over study. Subjects participating in the study will attend the clinical trial centre for screening, four treatment periods (Periods 1-4), and a follow-up visit (Figure 1); i.e. six visits over approximately 7-9 weeks.

General eligibility of subjects for participation in this study will be assessed at screening which will take place within 14 days of the first study drug administration. Subjects' eligibility regarding specific ECG criteria will be evaluated during screening.

In Periods 1 to 4, 32 eligible subjects will be randomised to 1 of 4 treatment sequences and receive single doses of E-52862 500 mg, or E-52862 600 mg, or E-52862 800 mg expressed as free base, or placebo. These sequences will provide balance for period and preceding treatment (Williams squares). Each period will consist of a baseline ECG day (Day -1) when subjects will receive placebo and a treatment day (Day 1) when subjects will receive either E-52862 or placebo, following a randomisation scheme. The ECG measurements on the baseline day will be taken at the corresponding clock time points as the ECG measurements and samples for PK analysis on the treatment day. There will be at least a 7-day washout interval between study drug administrations in Periods 1, 2, 3 and 4.

This study has been designed to explore the safety and tolerability of single doses of 500, 600 and 800 mg which exceed those investigated in an earlier Phase I dose escalation study specifically to provide assurance of the IMP's cardiac safety profile. A single episode of Atrial Fibrillation (AF), which reverted spontaneously, occurred in one healthy male volunteer after four once daily doses of 300 mg, however, this is considered very improbable to be drug related. Three days after stopping administration of E-52862, the subject experienced a new AF event which also reverted spontaneously; the subject was suffering from acute emotional stress when he experienced the two events and reported palpitations several months prior to entering the study. No relevant events on the 24-hour Holter ECG monitoring were observed in any of the 8 subjects treated daily for 8 days with 400 mg E-52862.

There were no preclinical findings suggestive of adverse cardiac effects. For the assessment of cardiac safety

formal investigations at the highest feasible exposure levels are deemed necessary to provide a degree of confidence during drug development. It would not be feasible to perform a TQT study at this juncture as both the therapeutic and the maximum tolerated dose (MTD) are currently unknown.

The study uses a multiple cross over design as this (a) allows dose escalation within the same person providing a greater margin of safety (b) allows the comparison of all test parameters within the same persons and (c) provides multiple baselines to perform thorough assessments of the ECG and cognitive effects.

Number of subjects:

Thirty two (32) healthy Caucasian males and females to be randomised for at least 28 subjects to complete all periods of the study. Of these subjects at least 8 subjects should be male or female.

Diagnosis and main criteria for admission:

Subjects will be included if they are male or female, Caucasian, 18 – 35 years (inclusive) of age, non smokers for at least 3 months, with a body mass index of 18 to 25 kg/m² inclusive, using an effective contraceptive method (or are abstinent), judged to be healthy from a medical history, physical examination, routine laboratory investigations and screening ECG assessments.

Main exclusion criteria are: any risk factor for the occurrence of Torsades de Pointes (marked baseline prolongation of QT/QTc interval: e.g. QTcB>450 msec, congenital or acquired long QT syndrome), any risk factor for the occurrence of AF, any pathology or abnormality or risk factor with possible influence on the ECG (electrolyte imbalance, impaired drug metabolism/clearance), the use of concomitant medications or foods that impair drug metabolising capacity, concomitant use of drugs with QT-prolonging effects.

All subjects included in the study must meet the ECG screening baseline selection criteria and be signed off for inclusion by a cardiologist.

Test treatment(s) and mode of administration

- A single oral dose of 500 mg E-52862.HCl, expressed as free base
- A single oral dose of 600 mg E-52862.HCl, expressed as free base
- A single oral dose of 800 mg E-52862.HCl, expressed as free base

Reference treatment(s) and mode of administration:

- A single oral dose of placebo matching E-52862

Duration of treatment:

A single oral dose of each study treatment per study period.

Criteria for evaluation:

<u>ECG analysis</u>

All ECG recordings will be examined for qualitative ECG variations, including morphological variations of the P wave, T wave, occurrence of a U wave, occurrence of ventricular arrhythmia and quantitative ECG variations including relative and absolute variations of mean QTc interval, relative and absolute variations of P and PR interval, relative and absolute variations of QRS interval.

Holter ECG analysis will include quantitative and qualitative descriptions of the assessment periods and a comparison to all off treatment assessments.

The baseline ECG recordings, at the beginning of each treatment period, are treatment and period specific and baseline ECG values are scheduled to match the “on-treatment” ECG sampling time points. All recordings are in triplicate and will be compliant with RPL’s SOPs for the correct recording of ECG (in thorough QT/QTc studies). Mean and median QT/QTc values will be calculated for each time point (triplicate ECG) for subsequent analyses.

The correction used for QTc will be the most accurate heart rate correction, chosen under blinding conditions by the statisticians from one of the following formulae using the all available “off treatment” ECG recordings [12-lead Holter ECG recordings at screening]:
(1) Individual correction (QTcI) (linear and nonlinear models); (2) Fridericia’s correction; (3) Bazett’s correction.

The primary baseline corrections will be calculated using averaged QTc baseline values (the mean of all median readings recorded for each time point on the baseline Day -1). This single value ($QTc^{baselineAV}$) will be used to calculate ΔQTc for each study period.

The effect on QTc will be calculated as the placebo subtracted time matched difference as:

$\Delta\Delta QTc = (QTc^{active}-QTc^{baselineAV})-(QTc^{placebo}-QTc^{baselineAV})$ calculated for each of the 12 post dose ECG time points.

© ESTEVE (2010) & CONFIDENCIAL

Statistical Methods

Descriptive analyses will be performed to appropriately qualify and quantify the finding from all ECG assessments. Summary statistics (n, arithmetic mean, median, minimum, and maximum) for all primary and secondary parameters will be calculated by treatment and time.

The analysis of interval changes will be based on the cross-over part of the study and will use the most appropriate heart rate correction (QTcI/QTcF/QTcB) and will be based on the change from average baseline. A linear mixed model with sequence, period, sex, time and time by treatment interaction as fixed effects, and baseline as covariate will be adapted, with subject as a random effect. Two-sided 90% confidence intervals for the difference between each dose of E-52862 and placebo will be derived.

Baseline will constitute the averaged baseline reference period used for the calculation of the baseline composite. The baselines are period specific in order to provide information on any possible carryover effects. Baseline ECG is scheduled to match the on-treatment clock time points for each treatment period.

Categorical analyses will be performed to determine the number of subjects per treatment regimen and time who had an increase from baseline QTc greater than or equal to 30 msec and greater than or equal to 60 msec. Individual subjects who have a QTc value greater than or equal to 450 msec, greater than or equal to 480 msec and greater than or equal to 500msec will be summarised for each treatment regimen by gender.

Scatter plots of QT and QTc against RR will be produced for all data to visualise the best correction formula. Plots of the differences with 90% CIs between all doses of E-52862 and placebo over time will be produced for all analyses to describe the concentration and effect relationship.

Cognitive tests

Computerised battery tests will be performed including:

- Groton maze learning task (executive function)
- One card learning task (working memory and learning)
- Detection task (simple reaction time/psychomotor function)
- Identification task (choice reaction time)
- Sustained vigilance test (repeat of the detection task)

A linear mixed model will be used to compare the treatment effect on cognitive test. The model will have sequence, period, sex, time and time by treatment interaction as fixed effects, baseline cognitive test as covariate, and subject as random effect. Two-sided 95 % confidence intervals for the difference between each dose of E-52862 and placebo at each time point will be derived.

Pharmacokinetic analysis

Blood for analysis of E-52862 levels in plasma will be collected at specified times for each period: All PK samples are taken after the corresponding ECG recordings.

The following endpoints will be determined for E-52862. They will be derived by non-compartmental analysis of the plasma concentration-time data (C_{max}, AUC, and $t_{½}$ values are assumed to be log-normally distributed):

- Maximum concentration (C_{max}).
- Time to reach maximum plasma concentration (t_{max}).
- Half-life ($t_{½}$).
- Area under the plasma concentration-time curve from zero to time t of the last measured concentration above the limit of quantification (AUC_{0-t}).
- Area under the plasma concentration-time curve from zero to infinity ($AUC_{0-\infty}$).

PK parameters will be analysed descriptively:

AUC_{0-t}, $AUC_{0-\infty}$, C_{max}, $t_{½}$, and t_{max} will be summarised with arithmetic mean, geometric mean, minimum, median, maximum, SD, standard error, CVb(%), and 95% confidence limits of the means for each dose group. Log-transformed AUC_{0-t}, $AUC_{0-\infty}$, C_{max}, and $t_{½}$ values will be summarised with geometric mean, SD of the logs, 95% confidence limits, and CVb(%).

Safety analysis

All emergent adverse events, laboratory investigations, physical and vital signs examinations (including pulse rate and blood pressure) will be included in the safety analysis.

© ESTEVE (2010) & CONFIDENCIAL

6. INTRODUCTION

Sigma (σ) receptors have been classified into two subtypes, σ_1 and σ_2, of which the σ_1 receptor is the only subtype that has been cloned so far (Guitart et al, 2004; Cobos et al, 2008). The endogenous receptor ligand is unknown although some peptides related to the neuropeptide and calcitonin gene-related peptide families which are known to display some affinity for σ_1 receptors, have been proposed to be the endogenous σ_1 receptor ligands (Maurice et al, 2006; Monnet et al, 2006). From a functional point of view, σ_1 receptors modulate the activity of a variety of receptors and ion channels and act as amplifiers in signal transduction cascades (Su et al, 2003). The σ_1 receptor is expressed in areas for pain control such as the spinal cord, the periaqueductal grey matter and the rostroventral medulla. Data supporting a role for σ_1 receptors in modulating pain has been restricted to studies describing a tonic inhibitory control of receptors on opiod receptor-mediated antinociception.

The concept that σ_1 receptors play a role in modulating pain behaviour in the absence of opiods, particularly in sensitising and chronic pain conditions (i.e. neuropathic pain), originated more recently from studies using genetic (σ_1 receptor knockout mice) and pharmacological approaches. Collectively, the results from these studies support a modulatory role of σ_1 receptors in spinal sensitisation and point to NMDA receptors and Ca^{2+}-dependent intracellular cascades as underlying mechanisms involved. This is not surprising as receptors are functionally coupled to NMDA receptors (Monnet et al, 1990; Martina et al, 2007) and regulate intracellular Ca^{2+} concentrations via phospholipase C and IP_3 receptors (Hayashi et al, 2000; Hayashi and Su, 2001; Su et al, 2003)). Hence, blocking of receptors is associated with reduced nerve injury-evoked activity of Ca^{2+-}permeable NMDA and IP_3 receptors, thus causing reduction of central sensitisation-related pain hypersensitivity.

Based on these studies, the σ_1 receptor is now considered a constituent of the mechanisms modulating activity-induced sensitisation in nociceptive pathways and thus a potential target for medicines designed to alleviate chronic pain.

E-52862 is a novel selective σ_1 receptor antagonist which has been investigated in *in vitro* and *in vivo* studies. The blocking of σ_1 receptors by E-52862 has shown effective analgesia in different models of chronic pain, particularly neuropathic pain as well as potentiating opioid analgesia in acute post-operative pain models.

6.1 Rationale for conducting study

The main purpose of this study is to assess the safety and tolerability of single ascending doses of E-52862 and to describe the cardiovascular safety profile and provide additional support for the use of E-52862 in patients with chronic pain including neuropathic pain, and during acute post-operative analgesia.

6.2 Risk-benefit evaluation

The healthy subjects will have no individual benefit from participating in this study. E-52862 is being developed primarily for the treatment of chronic pain and for the potentiation of opioid analgesia during acute post-operative pain based on a new mechanism of action. The benefit of these new drugs which are efficacious and well tolerated would be welcome treatments for patients with acute or chronic pain.

Potential risks to the study participants have been identified through internal and external reviews of the data obtained from pre-clinical and clinical studies conducted to date.

There is no safety margin for the proposed doses from animal data; doses given to humans to date have already exceeded no adverse effect plasma exposures in animals. Data from studies in humans however suggests that the proposed doses will be well tolerated; dose escalations are very conservative with escalations of 20-30% over the previous tolerated dose in the same individuals.

Central Nervous System (CNS) side effects are most likely to be dose limiting in a single ascending dose (SAD) study of E-52862. Convulsions occurred in very high doses in animal studies, however, there are no indications of CNS effects in clinical studies conducted to date and therefore convulsions are unlikely to be attained in this trial. There are no obvious markers for CNS effects however; cognitive function impairment will be measured as a biomarker for central pharmacological activity and safety.

The study will use a multiple cross-over design which will allow dose escalation in the same participants, starting with a dose that was previously well tolerated in healthy volunteers. Dose escalation will be sequential with placebo randomised into each escalation (Table 1). Monitoring of the hospitalised participants will be intense with continuous cardiac telemetry and thorough bed-side investigations.

Pre-clinical studies

E-52862 has been assessed in an extensive package of regulatory safety pharmacology studies. After oral administration in rats, no relevant effects on CNS (Irwin) and respiratory (plethysmography) functions were observed at the dose levels tested, while changes at the gastrointestinal (charcoal propulsion test) and renal functions were only observed at high dosages (reduced gastric emptying and urinary output, respectively). In terms of cardiovascular safety assessment, the *in vivo* dog cardiovascular telemetry study did not show any relevant effects on arterial blood pressure, heart rate or any of the lead II ECG parameters (PR, QRS, QT and QTcR intervals).

Pharmacokinetic data obtained to date for E-52862 shows good absorption in mice, rats, dogs and cynomolgus monkeys. The pharmacokinetic profile was found to be linear in mice in the dose range studied. The pharmacokinetic profile of E-52862 in rats and dogs appeared to be characterised by non-linear but dose-dependent kinetics over the dose range studied resulting in a higher systemic exposure than would be predicted from a linear relationship.

The toxicity of E-52862 has been studied following acute intravenous and oral administration in mice and rats, and after repeat oral dosing for up to 13 weeks in rats and dogs.

Following acute intravenous administration of E-52862, a dose of 50 mg/kg was considered to be the maximum non-lethal dose in mice and rats. After oral dosing, a maximum non-lethal dose of 1000 and 500 mg/kg in male and female mice, and 500 and 1000 mg/kg in male and female rats, respectively, was considered. The main signs of toxicity observed were consistent with CNS effects and included, decreased spontaneous activity, ataxia, ptosis, convulsions, loss of postural reflex, Straub phenomena and prostration.

After repeat oral administration in rats, the no observed adverse effect level (NOAEL) was considered to be 50 and 25 mg/kg/day after the 4 and 13-week treatment periods, respectively. Following repeat oral dosing in dogs, the NOAEL was considered to be 33 mg/kg/day after a 4-week treatment period and 18 mg/kg/day (males) and 5 mg/kg/day (female) after a 13-week treatment period. The majority of changes observed (see

investigator's brochure for further details) showed total or partial recovery after completing a treatment-free period.

The lowest NOAEL in animals (5 mg/kg/day) resulted in plasma levels of 308 ng/mL for C_{max} and 903 ng h/mL for $AUC_{0-\tau}$ compared to human plasma exposures of 4716.2 ng/mL fro C_{max} and 40732.9 ng h/mL for $AUC_{0-\tau}$ after a 500 mg single dose.

Clinical studies

A Phase I study with single oral doses of E-52862 has been completed and a second Phase I study with multiple oral doses has been completed.

E-52862 was administered as single escalating dose levels starting from 5 mg orally to healthy young male volunteers in the first-into-man study. This dose was subsequently increased in a stepwise manner up to a dose of 500 mg p.o. No serious adverse events (SAE) or deaths were observed, nor clinically relevant ECG, vital sign or laboratory values or central effects changes after administration of placebo or 5 to 500 mg of E-52862. The maximum tolerated dose (MTD) was not determined and the minimum tolerated dose (MID) was not reached. Dose levels lower or equal to 500 mg were considered adequately safe to be included during a subsequent multiple oral dose study.

The multiple oral doses study used 50 to 400 mg doses of E-52862 once-a-day for 8 days in healthy female and male volunteers. No SAEs or deaths were observed, nor clinically relevant vital sign or laboratory values or central effects changes after administration of placebo or E-52862. A single episode of Atrial Fibrillation (AF), which reverted spontaneously, occurred in one healthy male volunteer after four once daily doses of 300 mg, however, this is considered very improbable to be drug related: Three days after stopping administration of E-52862, the subject experienced a new AF event which also reverted spontaneously; the subject was suffering from acute emotional stress when he experienced the two events and reported palpitations several months prior to entering the study. No relevant events on 24-hour Holter ECG monitoring were observed in any of the 8 subjects treated daily for 8 days with 400 mg E-52862.

The pharmacokinetic data obtained to date indicates a good and rapid absorption of E-52862 in healthy human male volunteers.

The overall results of the single and multiple dose Phase 1 studies can then be listed as follows:

- E-52862 showed a good safety and tolerability profile after single and after multiple oral doses in healthy human volunteers.
- Adverse events were not dose dependent.
- There were no serious adverse events.
- No central effects were observed by means of ARCI (sedation, euphoria, dysphoria, intellectual efficiency and amphetamine effects) or VASs after administration of placebo or E-52862 at any dose.
- Maximum tolerated dose is at least 500 mg/day.
- Fast absorption was observed: t_{max} 0.75-1.38 h.

© ESTEVE (2010) & CONFIDENCIAL

- Elimination half-life: compatible with once-a-day administration.
- E-52862 C_{max} and AUC increases with dose.
- Parent (active compound) exposure is much higher than metabolites.
- The NOAELs described for rats and dogs might not a priori be interpreted as an indicator of the safety of E-52862 exposure in humans. E-52862 exhibits a significant higher metabolic stability in humans as compared to tested preclinical species. In rats and dogs, E-52862 is rapidly metabolised, i.e. the concentration of E-52862 is low and that of its metabolites is high. The opposite has been observed in humans, i.e. high concentration of E-52862 and low concentration of metabolites. Consequently, the parent (E-52862, active compound) / characterised metabolites ratio is significantly higher in human relative to rat and dog. Based on the preclinical and human data available to date, a hypothesis is that metabolites play a relevant role in the observed toxicology findings in preclinical species.

7. STUDY OBJECTIVES

7.1 Primary

- To assess the safety and tolerability of single ascending 500 mg, 600 mg and 800 mg doses of E-52862.

7.2 Secondary

- To describe the cardiovascular safety profile, including rhythm and conduction abnormalities, categorical QT/QTc interval data and qualitative and quantitative ECG variations from baseline.
- To describe and compare the number and the rates of adverse events under each treatment.
- To describe the pharmacokinetic profiles (PK) of E-52862 and metabolites in the study population.
- To describe effect response relationship, using a battery of cognitive tests.

8. STUDY DESIGN

8.1 Overall study design and procedures

This will be a single centre, double blind, randomised, placebo-controlled, four-way, cross-over study. Subjects participating in the study will attend the clinical trial centre for screening, four treatment periods (Periods 1-4), and a follow-up visit (Figure 1); i.e. six visits over approximately 7-9 weeks.

General eligibility of subjects for participation in this study will be assessed at screening which will take place within 14 days of the first study drug administration. Subjects' eligibility regarding specific ECG criteria will be evaluated during screening.

In Periods 1 to 4, 32 eligible subjects will be randomised to 1 of 4 treatment sequences and receive single doses of E-52862 500 mg, or E-52862 600 mg, or E-52862 800 mg, or placebo (Table 1). These sequences will provide balance for period and preceding treatment (Williams squares). Each period will consist of a baseline ECG day (Day -1) and a treatment day (Day 1). The ECG measurements on the baseline day will be taken at the corresponding clock time points as the ECG measurements and samples for PK analysis on the treatment day (Table 2). There will be at least a 7-day washout interval between study drug administrations in Periods 1, 2, 3 and 4.

This study has been designed to explore the safety and tolerability of single doses of 500 mg, 600 mg and 800 mg which exceed those investigated in an earlier Phase I dose escalation study (Investigator Brochure, version 4, 2010) specifically to provide assurance of the investigational medicinal product's (IMP) cardiac safety profile. A single episode of AF occurred in one healthy male volunteer after four once daily doses of 300 mg, however, this was considered very improbable to be drug related. There were no preclinical findings suggestive of adverse cardiac effects. For the assessment of cardiac safety formal investigations at the highest feasible exposure levels are deemed necessary to provide a degree of confidence during drug development (ICH E14, 2005). It would not be feasible to perform a TQT study at this juncture as both the therapeutic and the MTD are currently unknown.

The study uses a multiple cross-over design as this (a) allows dose escalation within the same person providing a greater margin of safety (b) allows the comparison of all test parameters within the same persons and (c) provides multiple baselines to perform thorough assessments of the ECG and cognitive effects.

Table 1 Study Flow Chart

Period 1		Washout*	Period 2		Washout*	Period 3		Washout*	Period 4	
**Day −1**	**Day 1**		**Day −1**	**Day 1**		**Day −1**	**Day 1**		**Day −1**	**Day 1**
Placebo	Placebo		Placebo	500 mg		Placebo	600 mg		Placebo	800 mg
Placebo	500 mg		Placebo	Placebo		Placebo	600 mg		Placebo	800 mg
Placebo	500 mg		Placebo	600 mg		Placebo	Placebo		Placebo	800 mg
Placebo	500 mg		Placebo	600 mg		Placebo	800 mg		Placebo	Placebo

E-52862 or Matching Placebo Capsule taken as a single oral dose in a sitting position with 240mL of water.

*Washout period will last for 7 days between doses (168 hours)

Table 2 Study Plan

Study Period	Screening	Period 1-4							Follow-Up
**Study Day**	**-14 to -3**	**-2**	**-1**	**1**	**2**	**3**	**4**	**5**	**7 to 14**
Informed Consent	X								
Demographic Data	X								
Medical History	X								
Physical Examination[a]	X	X							X
Inclusion / Exclusion Criteria[b]	X	X							
Body Weight / Height / BMI[c]	X	X							
Body Temperature	X								X
Blood Pressure / Pulse Rate	X	X[d]	X	X	X	X	X	X	X
Haematology / Biochemistry	X	X			X			X[e]	X
Serology	X								
Urinalysis	X	X						X[e]	X
Pregnancy Test for Females[f]	X	X							X
Urine Drugs of Abuse Screen[g]	X	X							
Alcohol Breath Test[h]	X	X							
Study Overnight Residence		X	X	X	X	X	X		
IMP Administration			X	X					
12-lead ECG[i]	X	X	X	X	X	X	X	X	
24 Hour 5-lead Holter ECG	X								
Telemetry ECG[j]			X	X					
Cognitive Testing[k]		X[l]	X	X	X				
PK Blood Sampling[i]				X	X	X	X	X	
PK urine collection[m]				X					
Adverse Events Recording	X	X	X	X	X	X	X	X	X
Concomitant Medication Recording	X	X	X	X	X	X	X	X	X

Wash-out between doses in each study period will be at least 7 days (144 hours); Follow-up 7-14 days after the last dose

a A complete physical examination will be performed at screening and follow-up. At admission on Day -2 of Period 1 only, a brief physical examination will be performed.

b Inclusion / Exclusion Criteria will be confirmed at Day -2 of Period 1 only.

c Body weight, height and BMI will be measured at admission on Day -2 of Period 1 only

d Day -2 of Period 1 only.

e Only measured at Day 5 of Period 4.

f Day -2 of Period 1 only.

g Day -2 of Period 1 only.

h Day -2 of Period 1 only.

i Timings are described in Table 3.

j 24-hour transmission and recording after dose on Day -1 and Day1 plus 1 hour pre-dose (25 hours).

k Performed at Days -1 and Day 1 as described in Table 3.

l Training sessions to avoid learning effects will be conducted on Day -2 or Period 1 only.

m PK urine collection from 0-12 hours on Day 1 only in Period 1.

n Meals will be served at standard Unit times as follows:
Day -2: dinner and snack; Day -1 and Day 1: lunch (5 hours post-dose), dinner (9 hours post-dose) and snack (13 hours post-dose)
Day 2 to 5: breakfast (24/48/72/96 hours post dose), lunch (5 hours post-breakfast), dinner (9 hours post breakfast) and snack (11.5 hours post-breakfast).

© ESTEVE (2010) & CONFIDENCIAL

Table 3 Assessment Time Schedule

Study Day	Protocol Time (hh:mm)	Dose	PK Blood Sampling	ECG	Cognitive Tests	Supine and standing BP/HR
**PERIODS 1 TO 4**						
**-2**				X	X[b]	X[e]
**-1**	Predose		X	X		X
	00:00	X				
	00:15			X		
	00:30			X		
	00:45			X		
	01:00			X		X[f]
	01:15			X		
	01:30			X		
	01:45			X		
	02:00			X	X[c]	X
	03:00			X	X[d]	X
	04:00			X		X
	06:00			X		X
	08:00			X		X
	12:00			X		X
**1**	Pre-dose (24:00)		X	X[a]		X
	00:00	X				
	00:15			X		
	00:30		X	X		
	00:45			X		
	01:00		X	X		X[f]
	01:15			X		
	01:30		X	X		
	01:45			X		
	02:00		X	X	X[c]	X
	03:00		X	X	X[d]	X
	04:00		X	X		X
	05:00		X			
	06:00		X	X		X
	08:00		X	X		X
	12:00		X	X		X
**2**	24:00		X	X	X	X
**3**	48:00		X	X		X
**4**	72:00		X	X		X
**5**	96:00		X	X		X

a In the statistical analyses, results of pre-dose ECG assessment on Day 1 will also be used for 24 hour baseline ECG assessments, as the two coincide.

b Two training sessions to be performed in Period 1 only.

© ESTEVE (2010) & CONFIDENCIAL

c To commence once the 2 hour 12-lead ECG recording has finished (to complete 15 minutes before the next) and once the 2 hour vital signs and PK measurements have finished.

d The sustained vigilance test will take place between the 3 and 4 hour post-dose time points.

e Day -2 of Period 1 only.

f At the 1 hour time point, standing blood pressure will not be measured.

8.1.1 Stopping Criteria

Group stopping criteria

After each period the available safety and tolerability data for E-52862 will be reviewed and assessed. This will include standard 12-lead ECGs (up to 24 hours post dose), 24-hour 5-lead ECG telemetry (alarms only), vital signs (up to 24 hours post dose), and clinical laboratory assessments (made on Day -2 and Day 2) and tolerability (including all adverse events).

The review will occur at the end of each period of each cohort thereby taking into account the safety information as it becomes available.

If E-52862 administration is considered sufficiently safe and tolerable, the decision to move to the next period will be made in agreement between Esteve and Richmond Pharmacology Ltd (RPL). Data will be distributed to a predefined audience by email with subsequent written confirmation of review, conclusions and resulting actions using a standard form for documentation purposes which will be included in the Study Operations Manual (SOM).

Predefined stopping criteria will regulate the escalating single dose process. Dose escalation will be discontinued either permanently or temporarily if any of the following dose-limiting events occur in the most recent dose period:

- Investigator and sponsor joint judgment of an unacceptable tolerability profile based on the frequency and intensity of observed AEs (including serious and related AEs). Further details will be defined in the SOM.
- If Grade II toxicity (using standard toxicity grading according to the National Cancer Institute Common Toxicity Criteria (NCI CTCAE version 4.0; local laboratory normal values are applied) is observed in two or more study subjects further dose escalation will be suspended and that dose level will not be repeated

  unless:

  a) The Grade II toxicity is clearly unrelated to the IMP or study procedures

  b) The Grade II toxicity is an expected drug effect due to the mode of action of the compound and does not represent a greater than the anticipated minimal safety risk to the subject(s)

  c) The Grade II toxicity is represented by commonly observed and therefore expected adverse events in healthy subject studies where a relationship to the study medication is unlikely but cannot be fully excluded and which do not present a significant clinical risk to the subjects such as pre-syncope, vasovagal reaction or anxiety
- Any occurrence of a possibly drug related Grade III or IV toxicity or a SAE will pause further dose escalation. The causal relationship to the IMP will be assessed and, if needed, the treatment will be identified (unblinding). If a causal relationship to the IMP is likely, then the trial will be suspended and the dosage level(s) will not be repeated.

© ESTEVE (2010) & CONFIDENCIAL

For the purpose of this study protocol, if any clinically relevant adverse events occur which in the reasonable opinion of the Investigator may require the pausing or discontinuation of the trial, then the following procedure to instigate an ad-hoc interim safety review will be followed:

1. The Sponsor will be informed immediately using the contact details specified in the SOM
2. Agreement will be reached between Sponsor and RPL to halt or continue further dosing and documented following the same process as the scheduled interim safety reviews

Any subject suffering from a clinically relevant adverse event will be monitored closely and provided with the appropriate medical care and follow up until the event has been resolved.

Individual stopping criteria

Dose escalation will be discontinued in a subject if any of the following dose-limiting events occur in the most recent dose period:

- Any subject experiencing Grade II toxicity will be withdrawn unless meeting the above exception criteria for Grade II toxicity.

For the purpose of this study protocol the term ‘suspension’ means that the dose level cannot be escalated or repeated in the next Period. Further dose may only take place after a substantial amendment has been submitted and approved by the Regulatory Authorities and the Research Ethics Committee.

8.2 Rationale for study design, doses and control groups

8.2.1 Choice of Subjects for Study

The study will be conducted in healthy male and female, Caucasian subjects to avoid interference from disease processes or other drugs. The selection criteria are defined such that subjects selected for participation in the study are known to be free from any significant illness and whose ECG allow a meaningful interpretation of results. Subjects will not derive any health benefits from participating in this study.

8.2.2 Dose Regimens of Administration

A single oral dose of E-52862.HCl (500 mg, 600 mg, 800 mg expressed as free base) or matching placebo will be administered in the fasted condition for each of the 4 study periods.

8.2.3 Justification of Doses

Pre-clinical animal toxicology studies are of limited predictive value since plasma level exposures found in humans exceed those seen in animal studies. In humans, C_{max} was found to be dose proportional with approximately 12.5 ng/ml/mg of E-52862 and the half-life in the order of 17 hours across a dose range of 5-500 mg. $AUC_{0-inf/D}$ was found to have a range of 55-80 (ng·h/ml)/mg with the highest values occurring after a 300 mg oral dose. In addition, taking into consideration the relatively long plasma half-life, drug plasma levels after chronic dosing exceed those seen after a single dose.

Clinically, E-52862 has been generally well tolerated in human studies conducted to date. Anticipated side effects from a single dose are unknown. At single doses of up to 500 mg

© ESTEVE (2010) & CONFIDENCIAL

and chronic doses up to 400 mg over eight days (resulting in higher exposure levels) no trend was observed. It therefore seems reasonable to further escalate single doses to 600 mg and 800 mg in a sequential manner. Furthermore, because of the sensitivity of cognitive measurements, it also seems reasonable to assess cognitive performance using a battery of computerised tests.

A single dose design was chosen as higher peak plasma concentrations are thought to be the most useful for the clinical assessment of potential cardiotoxicity.

8.2.4 Monitoring and Communication of Adverse Events / Adverse Drug Reactions

AEs will be continuously monitored throughout the study from the date informed consent is signed until the end of subject's participation. Each AE reported will be assessed by a trained Research Physician who will ensure that the event is dealt with as appropriate based on clinical need, study protocol and the clinical pharmacology unit's (CPU) standard operating procedures (SOPs). AEs will be documented in the subjects' Case Report Forms (CRFs) and reviewed regularly by the Research Physicians and the Investigator.

If any information relating to the study drug in this study becomes available after the submission of a final protocol to the Competent Authority which may impact on the conduct of the study, including but not limited to the risk and benefit evaluations underpinning approvals and volunteer's consent, Esteve shall notify RPL in writing as soon as practically possible and the parties will agree, in writing, what steps need to be taken if any.

8.2.5 Investigator Site Facilities and Personnel

This study will be conducted in a specialised early phase CPU within an acute hospital setting with Critical Care facilities, thus ensuring direct access to equipment and staff for resuscitating and stabilising subjects in acute medical conditions and emergencies. The study is conducted by an experienced Principal Investigator and well trained medical, nursing and technical staff with ample experience in the conduct of early phase clinical trials.

The study is designed to closely monitor, treat and communicate potential expected adverse reactions (based on the known mode of action of the IMP and the previous studies) as well as potential unexpected AEs.

9. SELECTION AND WITHDRAWAL OF SUBJECTS

9.1 Number and Source of Subjects

Thirty two (32) healthy Caucasian males and females to be randomised for at least 28 subjects to complete all periods of the study. Of the randomised subjects at least 12 subjects (approximately 40%) should be male or female.

9.2 Inclusion Criteria

A subject will be eligible for inclusion in this study only if all of the following criteria apply:

1. Healthy male or female subjects aged between 18 and 35 years (inclusive) at screening.
2. Signed informed consent in the local language prior to any study-mandated procedure.

3. No clinically significant findings on the physical examination at screening and at admission on Day −2 of Period 1.
4. Body mass index (BMI) between 18 and 25 kg/m^2 (inclusive) at screening and at admission on Day −2 of Period 1, body weight at least 50 kg.
5. Systolic blood pressure 90–130 mmHg, diastolic blood pressure 50–80 mmHg, and pulse rate 40–90 bpm (all inclusive), measured on the left arm, after 10 minutes in the supine position at screening and at admission on Day -2 of Period 1.
6. Triplicate 12-lead ECG without clinically relevant abnormalities measured after 10 minutes in the supine position at screening and at admission on Day -2 of Period 1.
7. 24-hour 5-lead Holter ECG without clinically relevant abnormalities measured at screening.
8. Haematology, biochemistry, and urinalysis test results not deviating from the normal range to a clinically relevant extent at screening and at admission on Day -2 of Period 1.
9. The Caucasian subjects should be distinguished especially by very light to brown skin pigmentation and straight to wavy or curly hair, and should be indigenous to Europe, northern Africa, western Asia, and India. Therefore, the study may as well include Caucasian subjects from North America, Australia and South Africa.
10. Subjects must agree to use acceptable methods of contraception:

    <u>Female subjects</u>

    Female subjects of childbearing potential must use medically acceptable methods of contraception from the time of the first administration of the study medication until three months following administration of the last dose of study medication. Acceptable methods include:

    - A documented placement of an intrauterine device (IUD) or intrauterine system (IUS) and the use of a barrier method {condom or occlusive cap (diaphragm or cervical/vault caps) used with spermicidal foam/gel/film/cream/suppository};
    - Documented tubal ligation (female sterilisation). In addition, a barrier method {condom or occlusive cap (diaphragm or cervical/vault caps) used with spermicidal foam/gel/film/cream/suppository} should also be used;
    - Double barrier method: Condom and occlusive cap (diaphragm or cervical/vault caps) with spermicidal foam/gel/film/cream/suppository;
    - Abstinence.

    <u>Male subjects</u>

    Male subjects must use medically acceptable methods of contraception if their female partner(s) is (are) pregnant from the time of the first administration of the study medication until three months following administration of the last dose of study medication. Acceptable methods include:

    - Condom,
    - If the subject has undergone surgical sterilisation (vasectomy with documentation of azoospermia) a condom should also be used.

    Use acceptable methods of contraception if the male subject’s partner could become pregnant from the time of the first administration of study medication until three months

© ESTEVE (2010) & CONFIDENCIAL

following administration of the last dose of study medication. The acceptable methods of contraception are as follows:

- Condom and occlusive cap (diaphragm or cervical/vault caps) with spermicidal foam/gel/film/cream/suppository;
- Surgical sterilisation (vasectomy with documentation of azoospermia) and a barrier method {condom or occlusive cap (diaphragm or cervical/vault caps) used with spermicidal foam/gel/film/cream/suppository};
- The female partner uses oral contraceptives (combination oestrogen/progesterone pills), injectable progesterone or subdermal implants and a barrier method {condom or occlusive cap (diaphragm or cervical/vault caps) used with spermicidal foam/gel/film/cream/suppository};
- Medically prescribed topically-applied transdermal contraceptive patch and a barrier method {condom or occlusive cap (diaphragm or cervical/vault caps) used with spermicidal foam/gel/film/cream/suppository};
- The female partner has undergone documented tubal ligation (female sterilisation). In addition, a barrier method {condom or occlusive cap (diaphragm or cervical/vault caps) used with spermicidal foam/gel/film/cream/suppository} should also be used;
- The female partner has undergone documented placement of an intrauterine device (IUD) or intrauterine system (IUS) and the use of a barrier method {condom or occlusive cap (diaphragm or cervical/vault caps) used with spermicidal foam/gel/film/cream/suppository};
- Abstinence.

11. Ability to communicate well with the Investigator in the local language, and to understand and comply with the requirements of the study.

9.3 Exclusion Criteria

A subject will not be eligible for inclusion in this study if any of the following criteria apply:

1. History or clinical evidence of any disease and/or existence of any surgical or medical condition which might interfere with the absorption, distribution, metabolism or excretion of the study drug (appendectomy and herniotomy allowed, cholecystectomy not allowed).
2. History of clinically significant syncope.
3. Family history of sudden death.
4. Family history of premature cardiovascular death.
5. Clinically significant history or family history of congenital long QT syndrome (e.g. Romano-Ward syndrome, Jervell and Lange-Nielson syndrome) or Brugada's syndrome.
6. History of clinically significant arrhythmias and ischemic heart disease (especially ventricular arrhythmias, atrial fibrillation, recent conversion from atrial fibrillation or coronary spasm).
7. Conditions predisposing the volunteer to electrolyte imbalances (e.g. altered nutritional states, chronic vomiting, anorexia nervosa, bulimia nervosa).

© ESTEVE (2010) & CONFIDENCIAL

8. ECG abnormalities in the standard 12-lead ECG (at screening and Day -2 of Period 1) and 24-hour 5-lead Holter ECG (at screening) which in the opinion of the Investigator will interfere with the ECG analysis.
9. Any clinically important abnormalities in rhythm, conduction or morphology of resting ECG that may interfere with the interpretation of QTc interval changes. This includes subjects with any of the following (at screening and Day -2 of Period 1):
   - Sinus node dysfunction.
   - Clinically significant PR (PQ) interval prolongation.
   - Intermittent second or third degree AV block.
   - Incomplete or complete bundle branch block.
   - Abnormal T-wave morphology.
   - Prolonged QTcB >450 msec or shortened QTcB <350 msec or family history of long QT syndrome.

   Subject with borderline deviations from these criteria may be included if the deviations do not pose a safety risk, and if agreed between the appointed Cardiologist and the Principal Investigator.
10. Signs and/or symptoms of a clinically relevant acute illness in the 4-week period prior to screening.
11. Veins unsuitable for intravenous puncture on either arm (e.g., veins that are difficult to locate, access or puncture, veins with a tendency to rupture during or after puncture).
12. Known hypersensitivity to any medicines administered in the trial.
13. Treatment with any prescribed medication during the two weeks prior to first baseline day.
14. Treatment with any over-the-counter (OTC) medications during the two weeks prior to first baseline day.
15. Treatment with vitamins and/or minerals within 48 hours prior to the first baseline day.
16. Treatment with another investigational drug within three months prior to dosing or having participated in more than three investigational drug studies within 1 year prior to dosing.
17. Confirmed positive results from urine drug screen (amphetamines, benzodiazepines, cocaine, cannabinoids, opiates, barbiturates and methadone) or from the alcohol breath test at screening and on Day −2 of Period 1.
18. History or clinical evidence of alcoholism or drug abuse. Alcohol abuse is defined as regular weekly intake of more than 14 units if female and 21 units if male; drug abuse is defined as compulsive, repetitive and/or chronic use of drugs or other substances with or without problems related to their use and/or where stopping or a reduction in dose will lead to withdrawal symptoms.
19. Excessive caffeine consumption, defined as ≥ 800 mg per day at screening (800 mg = 7 cups of coffee or 16 cups of tea).
20. Chronic use of tobacco (smoking, snuff) or other nicotine containing products (nicotine chewing gum, nicotine plaster or other smoking cessation therapy) for at least three months.
21. Loss of 250 mL or more blood within three months prior to screening.

© ESTEVE (2010) & CONFIDENCIAL

22. Positive results from the hepatitis serology, except for vaccinated subjects, at screening.
23. Positive results from the HIV serology at screening.
24. Any circumstances or conditions, which, in the opinion of the Investigator, may affect full participation in the study or compliance with the protocol.
25. Legal incapacity or limited legal capacity at screening.

9.4 Subject Restrictions

Subjects will have to comply with the following restrictions:

Standardised meals will be consumed during the study period at the investigational site as detailed in Table 2. Should other assessments coincide with meals, the meals will commence after these assessments (e.g. ECG and PK) have been completed at each respective time point.

Before the administration of investigational products (Periods 1 to 4), the subjects will fast overnight for at least 10 hours. Except for water used for dosing, fluids will not be allowed from 1 hour before oral dosing until after 2 hours post-dose.

For all Clinical Pharmacology Unit days, menus (including all food items), indicating calories (kcal), protein (in g), fat (in g) and carbohydrates (in g) for each meal will be documented in the site study file. All meals and snacks consumed in the unit will not contain enzyme-inducing constituents (e.g. broccoli, brussel sprouts, cabbage, etc.).

Intake of alcohol will not be allowed from 72 hours prior to each baseline ECG day and during each visit at the investigational site (including the screening and follow-up visit as well as the study days).

Smoking is not allowed for the whole duration of the study.

The subjects will have to refrain from grapefruit, grapefruit juice, Seville oranges, watercress, broccoli, brussel sprouts, or cabbage intake from seven days before the baseline ECG day in Period 1 until after the last study period.

Intake of caffeine will not be allowed from 48 hours prior to each baseline ECG day until discharge from the unit in each period.

Blood donation will not be allowed at any time during the study and up to three months after completion of the study.

The subjects must refrain from strenuous physical exercise from 48 hours before screening until after follow-up.

The subjects must abstain from consumption of energy drinks containing taurine or glucuronolactone from 72 hours before admission until the follow-up visit.

For restrictions regarding concomitant medication please refer to Section 10.6.

© ESTEVE (2010) & CONFIDENCIAL

9.5 Subject inclusion and randomisation

Subjects who sign the informed consent for the study and thereafter undergo screening procedures will be identified during this period by their unique RPL ID number (identification number from the RPL volunteer database and screening number).

Subjects fulfilling the eligibility criteria will be randomised and assigned subject numbers on the morning of Day -1 in Period 1. Subjects will be assigned numbers 01 to 32.

Subject numbers will be allocated in consecutive order and correspond to a number on the computer generated randomisation list, which determines the treatment sequence.

If a subject discontinues from the study, the subject number will not be re-used and the subject will not be allowed to re-enter the study.

9.6 Randomisation

A randomisation list, detailing which sequence the IMPs are to be administered to each study subject, will be generated by RPL's statistician using SAS™ PROC PLAN. The randomisation list will be kept in a locked cabinet at RPL's Pharmacy.

Blocked randomisation with a block size of 4 will be performed. For Periods 1 to 4, randomisation code will be generated according to a 4 x 4 Williams square.

9.7 Withdrawal of Subjects

9.7.1 Criteria for withdrawal

Subjects may be withdrawn at any time but once dosing has occurred every attempt should be made to continue assessments to ensure the safety of the subject. Specific reasons for withdrawing a subject may be:

- Voluntary discontinuation by the subject, who is at any time free to discontinue his/her participation in the study.
- Safety reasons as judged by the Investigator and/or Esteve (See Section 8.1.1).
- Severe non-compliance to protocol as judged by the Investigator and/or Esteve (e.g., positive pregnancy test or positive results from any urine drug screen or positive alcohol breath test).
- Incorrect inclusion, i.e. the subject, with the benefit of hindsight, did not meet the required eligibility criteria for the study at the time of inclusion.
- Other reasons as judged by the Investigator.

9.7.2 Procedures for subject withdrawal

Subjects are at any time free to withdraw from the study, without prejudice (withdrawal of consent). Such subjects will always be asked about the reason(s) and the presence of any

© ESTEVE (2010) & CONFIDENCIAL

AEs. If possible, subjects who withdraw from the study after dosing and before completion should be seen by an Investigator or delegate and undergo the assessments and procedures scheduled for the follow-up visit. AEs should be followed up for a sufficient time according to Investigator/Sponsor criteria).

10. STUDY AND CONCOMITANT TREATMENTS

10.1 Investigational Medicinal Products (IMPs)

The following four treatments will be administered to all subjects (randomised for sequence) in the study. The E-52862 hard gelatin capsules to be used in this study will be single strength containing 100 mg as free base or 200 mg as free base or 300 mg as free base.

- One single dose of 500 mg E-52862.HCl expressed as free base.
- One single dose of 600 mg E-52862.HCl expressed as free base.
- One single dose of 800 mg E-52862.HCl expressed as free base.
- One single dose of placebo matching E-52862.

Study Period			
**Treatment Group:**	**Treatment:**	**IMP Dispensed[a]:**	**Administration**
A	500 mg E-52862	2 x $E\text{-}52862^{200}$ + 1 x $E\text{-}52862^{100}$	One oral single dose of Treatment D on Day -1 of each period and Treatments A, B, C or D each on Days 1 in Periods 1 to 4 per randomisation schedule administered with 240 mL of still water
B	600 mg E-52862	3 x $E\text{-}52862^{200}$	
C	800 mg E-52862	2 x $E\text{-}52862^{300}$ + 1 x $E\text{-}52862^{200}$	
D	Placebo	3 x $P^{E\text{-}52862}$	

[a] the number of tablets for each IMP are shown.

Esteve will take care of the study medication preparation, including labelling and packaging. Richmond Pharmacology will receive individual medication boxes, properly labelled.

10.1.1 Doses and treatment regimen

Oral doses will be administered by a Research Physician between 08:00 a.m. and 10:00 a.m. and the details of dosing will be recorded in the CRF. The dosing will be verified by another member of the Investigator's staff. Oral doses will be administered with approximately 240 mL of water at room temperature (extra water will be allowed if needed to swallow the capsules, upon the Investigators criterion). Before the administration of investigational products the subjects will fast overnight for at least 10 hours. Except for water used for dosing, fluids will not be allowed from 1 hour before oral dosing until after 2 hours post-dose.

© ESTEVE (2010) & CONFIDENCIAL

10.2 Labelling of Investigational Medicinal Products

Esteve will take care of the study medication preparation, including labelling and packaging: Richmond will receive individual medication boxes, properly labelled

The labels will fulfil Good Manufacturing Practice (GMP) Annex 13 requirements for labelling. All labelling will be prepared in accordance with GMP and local regulatory guidelines.

10.3 Drug Accountability

The Investigator or pharmacy staff is responsible for correct storage of the trial medication according to the manufacturer’s recommendations. The trial medication made available for this clinical trial must be used in accordance with the protocol and must only be handled by the pharmacist or authorised personnel. The pharmacy staff must maintain complete and accurate records, showing the receipt and disposition of all supplies of the trial medication. These records must include a master record which lists the date of receipt of all trial medication and the quantities received, and a dispensing record which includes all quantities dispensed, subject numbers to whom trial medication is dispensed, the date of each dispensing, and the identification of the dispenser. After the Clinical Research Associates (CRAs) designated by Esteve have checked and approved the drug accountability, all unused medication will sent to Esteve who will arrange destruction.

10.4 Storage

No special storage conditions are required.

10.5 Blinding and Procedures for Unblinding the Study

10.5.1 Methods for ensuring blinding

This study is double-blind (both subject and Investigator are blinded) with regard to administration of investigational products (received on Day 1). Only subjects will be blind with regard to medication received on Day -1.

10.5.2 Methods for unblinding the study

Individual treatment codes, indicating the treatment randomisation for each randomised subject, will be available to the Investigator(s) or pharmacists at the study centre. Treatment codes will be kept in a locked cabinet at RPL’s Pharmacy. The individual treatment codes must not be broken except in medical emergencies when the appropriate management of the subject necessitates knowledge of the treatment randomisation. The Investigator must document and report to Esteve any breaking of the treatment code. Esteve retains the right to break the code for SAEs suspected to be causally related to an IMP and that potentially require expedited reporting to regulatory authorities.

10.6 Concomitant Medications

The subjects should refrain from use of prescribed medication during the two weeks before the baseline ECG day in Period 1 and use of OTC drugs (including herbal remedies and

minerals) in the two weeks before the baseline ECG day in Period 1 until the last follow-up visit. Use of vitamins is not allowed within 48 hours before the ECG baseline day in Period 1 until the last follow-up. Subjects should also refrain from use of any enzyme affecting drugs and/or any herbal remedies which may affect the enzymes from 14 days before the baseline ECG day in Period 1 until the last follow-up. Occasional use of paracetamol (up to 2 g/day) for mild pain such as headache relief is allowed. If concomitant medication is taken the Investigator must decide if the healthy volunteer should remain in the study or be withdrawn.

Details of previous and concomitant treatments should be recorded throughout the study by the Investigator on the appropriate pages of the CRF.

10.7 Treatment compliance

Oral doses will be administered by a qualified member of staff as per RPL's SOP and the details of dosing will be recorded in the CRF. The procedure should preserve blinding of subjects to medication in both Day -1 and Day 1. The dosing will be verified by another member of the Investigator's staff. Oral doses will be administered with 240 mL of water at room temperature.

The administration of all medication (including investigational product) should be recorded in the appropriate sections of the CRF. The dose, date and time of administration of the investigational product will be recorded and checked by the monitor at monitoring visits.

11. STUDY PROCEDURES

11.1 Schedule of Study Procedures

The study will consist of a screening visit, study period (Periods 1 to 4), and a follow-up visit. There will be one in-house period from Day -2 of Period 1 to Day 5 of Period 4. There will be an interval of at least 7 days (168 hours) between the doses in each period and a follow-up visit 7-14 days after the last dose of study drug. The study will last up to approximately 49 days in total, including the screening and follow-up visits. AEs should be collected and recorded for each subject from the date the Informed Consent Form (ICF) was signed until the end of their participation in the study.

The priority order for assessments at a particular time point (post-dose) is:

ECG recording

Vital signs

PK blood sample

11.1.1 Screening visit

Day -14 to Day -3

Each subject will undergo a screening assessment within two weeks before the start of the study period phase. The start of the study period phase is defined as the day on which subjects received their first randomised dose of either E-52862 500 mg, E-52862 600 mg,

© ESTEVE (2010) & CONFIDENCIAL

E-52862 800 mg, or placebo. The Principal Investigator will obtain a written ICF from the subject prior to any study related procedures being performed. The screening will consist of:

- Informed consent
- Inclusion/exclusion criteria
- Recording of demographic data - date of birth, gender, height, weight, BMI, race
- Standard medical/surgical history and physical examination including general appearance, skin, head and neck, lymph nodes, thyroid, abdomen, musculo-skeletal, cardiovascular, respiratory and neurological system
- Vital signs measurement (supine/standing BP and pulse rate) and body temperature
- 12-lead ECG recording (measured in triplicate)
- Ambulatory 5-lead Holter (24 hours)
- Haematology and Biochemistry
- Serology assessments including Hepatitis B surface antigen, Hepatitis C antibodies, and HIV 1 and 2 antibodies
- Urine pregnancy test for females
- Urinalysis
- Urine drugs of abuse screen
- Alcohol breath test
- AE and concomitant medication check

Should there be any clinically significant abnormalities in the results of these examinations, RPL will inform the subject’s general practitioner as appropriate, unless it is an emergency in which case the subject would immediately be referred to a specialist.

Subjects fulfilling the protocol inclusion/exclusion criteria will be invited to participate in the study.

11.1.2 Study Periods 1 to 4

Day -2 (Admission)

Subjects will arrive at the Unit in the afternoon of Day -2 of Period 1 and remain resident at the Unit until the morning of Day 5 of Period 4. Inclusion/exclusion criteria will be confirmed at Period 1 only.

Physical examination, body weight, height and BMI (measured at Period 1 only), 12-lead ECG recording (measured in triplicate), Vital signs measurement (Period 1 only), Haematology and Biochemistry, urine drugs of abuse screen and alcohol breath test (Period 1 only), urinalysis, will be performed for all subjects as well as a urine pregnancy test for

© ESTEVE (2010) & CONFIDENCIAL

females (Period 1 only). An AE and concomitant medication check will be performed. Training sessions for the cognitive tests will also be conducted on Day -2 to avoid learning effects.

Dinner and a snack will be served at standard Unit time.

Day -1 (Baseline)

All subjects will be administered placebo (matching E-52862) in order to establish a baseline for ECG recordings. Placebo will be administered as a total of 3 capsules with 240 mL of water following an overnight fast of at least 10 hours.

ECG recordings (measured as triplicates) will be performed at the time points detailed in Table 3.

25 hour continuous telemetry (1 hour pre-dose) will be performed after dosing.

Supine/standing BP and pulse rate will be recorded at the time points detailed in Table 3.

Cognitive testing will be performed at the time points detailed in Table 3.

An AE and concomitant medication check will be performed.

Subjects will be served meals as described in Table 2.

Day 1

All subjects will be administered their first randomised dose of either E-52862 500 mg, E-52862 600 mg, E-52862 800 mg or matching placebo for E-52862. The dose will be administered as a total of 3 capsules with 240 mL of water following an overnight fast of at least 10 hours.

ECG recordings (measured as triplicates) will be performed at the time points detailed in Table 3.

25 hour continuous telemetry (1 hour pre-dose) will be performed after dosing.

Supine/standing BP and pulse rate will also be recorded at the time points detailed in Table 3.

Cognitive testing will be performed at the time points detailed in Table 3.

PK blood sampling and urine collection will be performed at the time points detailed in Table 2 and Table 3.

An AE and concomitant medication check will be performed.

Subjects will be served meals as described in Table 2.

Note: In the statistical analyses, results of pre-dose ECG assessment on Day 1 will also be used for 24 hour baseline ECG assessments, as the two coincide.

Days 2-4

ECG recordings (measured as triplicates), supine/standing BP and pulse rate, and PK blood sampling will be performed and recorded at 24 hours, 48 hours, and 72 hours post dose. PK urine collection will be performed at the time points detailed in Table 2 and Table 3. Blood sampling for haematology and biochemistry will be taken (Day 2 only). Subjects will be served meals as described in Table 2. An AE and concomitant medication check will be performed. Cognitive testing will be performed once the other assessments are finalised, as detailed in Table 3.

Day 5

ECG recordings (measured as triplicates), supine/standing BP and pulse rate, and PK blood sampling will be performed/recorded at 96 hours post dose. Breakfast will be served. An AE and concomitant medication check will be performed.

Following an assessment by a Research Physician, all subjects will be discharged from the Unit in the morning of Period 4. There will be a washout interval of at least 7 days between Day 1 study drug administrations in Periods 1 to 4.

11.1.3 Follow-up

Subjects will attend the follow-up assessments within 7-14 days after the last dose of the study drug when they will undergo a physical examination including body temperature, pulse rate, supine/standing BP measurement, and safety laboratory tests (haematology, biochemistry, urinalysis, and urine pregnancy test for females). An AE and concomitant medication check will be performed.

11.2 Recording of data

The Principal Investigator will ensure that data are recorded on the paper CRFs (pCRF) as specified in the study protocol. He will ensure the accuracy, completeness, and timeliness of the data recorded, for data queries and all required reports according to any instructions provided. An AE and concomitant medication check will be performed.

The Principal Investigator will sign the completed CRFs. A copy (scanned) of the completed CRFs will be archived by an external service provider.

12. STUDY METHODOLOGY

All study measurements to be obtained are described below. An overview of these measurements is given in the study plan (Table 2). Timings of the measurements are detailed in Table 3. The following ‘priority order’ will be in effect when more than one assessment is required at a particular time point: ECG recording, BP/ pulse rate, PK blood sampling, and safety laboratory. Pre-dose assessments may be performed up to 120 minutes prior to dosing.

Should there be any safety concerns based on a review of the safety data for an individual subject, additional vital signs, ECG recordings, and/or laboratory safety samples may be taken. Additional PK samples per study period may also be taken if deemed necessary but the total volume of blood to be withdrawn during the study will not exceed 550 mL.

12.1 Laboratory Safety Measurements

12.1.1 Haematology, biochemistry

Blood samples for determination of biochemistry and haematology parameters will be taken at the times given in the study plan (Table 2). The date and time of collection will be recorded on the appropriate CRF. The analyses will be done at The Doctors Laboratory (TDL), using routine methods.

Blood samples for determination of standard haematology parameters will be collected in 4 mL EDTA tubes and blood samples for determination of standard biochemistry parameters will be collected in 5 mL SST tubes.

Laboratory values outside the reference limits, which are suspected to be of any clinical significance, will be repeated. Subjects in whom the suspected clinical significance is confirmed on repeated sampling will either not be included or, if already included, may be withdrawn from further participation in the study and/or followed until normalisation or for as long as the Investigator considers necessary.

Laboratory parameters to be measured are presented in Table 4 and the total amount of blood to be taken during the study is given in Table 5.

12.1.2 Serology

At the screening visit all subjects will be tested for Hepatitis B surface antigen, Hepatitis C antibodies, and HIV 1 & 2 antibodies. This is done for the safety of the study personnel and the result from the tests will not be entered into the study database. If a subject is found positive to any of these tests, he or she will be referred for further examination and treatment and will not be included in the study. The samples will be analysed by TDL.

12.1.3 Urinalysis

Urine samples for determination of urinalysis parameters will be taken at the times given in the study plan (Table 2). Urinalysis will be performed by RPL using a dipstick method, which provides information regarding leukocytes, nitrite, urobilinogen, protein, pH, blood, specific gravity, ketones, bilirubin, and glucose in/of the urine. If deemed necessary, based on a clinically significant positive test, microscopic examination of sediment and/or culture will be performed by TDL.

12.1.4 Pregnancy test

To exclude pregnancy, a urine pregnancy test will be performed at screening, admission (Day -2 of Period 1 only (Table 2)). A urine pregnancy test will be performed whenever pregnancy is suspected. Any subject with a positive pregnancy test will be excluded or withdrawn.

12.1.5 Drugs of abuse

Urine will be tested for the following drugs of abuse: benzodiazepines, opiates, amphetamines, methadone, cocaine, cannabinoids, and barbiturates at RPL (for details see

© ESTEVE (2010) & CONFIDENCIAL

Table 2). If a subject fails the drug abuse screen, they will be excluded from the study. A repeat drug screen can only be done where methodological reasons are believed to have led to a false positive. Borderline positive results, unless covered by the preceding condition, are to be considered as positive and the subject excluded from the study. If subjects are found to be positive due to medication e.g. flu/cold remedies they may undergo a repeat drug screen if they are still within the screening window. The results from the tests will not be entered into the database.

12.1.6 Physical examinations

The timing of individual examinations is indicated in the Study Plan (see Table 2). A complete physical examination will be performed at screening and follow-up and include an assessment of the following: general appearance, skin, head and neck, lymph nodes, thyroid, abdomen, musculo-skeletal, cardiovascular, respiratory and neurological systems.

On admission prior to dosing only a brief physical examination is required and will include assessment of the following: general appearance, skin, head and neck, abdomen, cardiovascular, respiratory and neurological systems (other systems may be reviewed if appropriate).

Height (screening and Day -2 of Period 1 only) will be measured in centimetres and weight in kilograms. Measurements should be taken without shoes and using calibrated scales for all measurements. BMI will be calculated from the height and weight.

Table 4 Laboratory Safety Variables that will be Measured

**Clinical chemistry**	**Haematology**
Aspartate aminotransferase	Red blood cells
Alanine aminotransferase	Haemoglobin
Alkaline phosphatase	Haematocrit
Lactate dehydrogenase	Mean corpuscular volume
Creatine kinase	Mean corpuscular haemoglobin concentration
Gamma GT	White blood cells
Total bilirubin	Neutrophils
Creatinine	Lymphocytes
Urea	Monocytes
Total Protein	Eosinophils
Albumin	Basophils
Glucose	Platelet count
Sodium	
Potassium*	**Urinalysis**
Calcium	Leukocytes
Phosphate	Nitrite
Cholesterol	Urobilinogen
Triglycerides	Protein
Direct bilirubin	pH
	Blood
**Serology**	Specific gravity
Hepatitis B surface antigen	Ketones
Hepatitis C antibodies	Bilirubin
HIV 1 and 2 antibodies	Glucose

*Samples from screening, from Day -2 (Periods 1 to 4) are used for safety assessment only.

12.2 Alcohol Breath Test

An alcohol breath test will be done (for details see Table 2), using an alcometer. The results from this test will not be entered into the clinical study database. This test will be carried out at screening and admission (Day -2) for Periods 1 only. It may also be performed randomly during the study. If a subject tests positive to the test they will be excluded from the study.

12.3 Vital Signs

12.3.1 Blood pressure and pulse rate

Supine/standing BP and pulse rate will be measured using a semi-automatic BP recording device (Dinamap® monitors) with an appropriate cuff size. The subjects will be required to rest in a supine position for at least ten minutes prior to BP and pulse measurements and for one minute before each standing measurement. For timings of individual measurement refer to the Study Plans (Table 2 and Table 3).

12.3.2 Body temperature

Body temperature (tympanic) will be measured (a single measurement) in degrees Celsius using an automated thermometer at the times indicated in the Study Plan (Table 2). Additional temperature assessments may be taken for safety at the discretion of the Principal Investigator or delegate.

12.4 ECG Measurements

12.4.1 Recording of 12-lead ECG

12-lead ECG will be recorded using a MAC1200® recorder connected via a fixed network connection to the MUSE® Cardiology Information System (MUSE). All ECG recorded during the study will be stored electronically on the MUSE information system. Only ECG recorded electronically will be valid ECG for any purpose other than safety assessment. ECG printouts may be filed in the subject's CRF for medical safety reviews.

If at all possible, the same recorder will be used for any one subject. Each ECG recorder will be set up to the required technical specifications and containing the information required to identify the records. Each ECG recording will be clearly identified (Subject ID, scheduled time relative to dose, and the actual times of ECG recordings).

12-Lead ECG recordings will be made at the time points indicated in the study plan and time schedule (Table 2 and Table 3) after the subjects have been resting in a supine position for at least 10 minutes. The subjects will avoid postural changes during the ECG recordings and clinical staff will ensure that subjects are awake during the ECG recording. The use of a semi permanent skin marker will ensure consistent placement of the leads.

At each time point, the ECG will be recorded in triplicate, to reduce variance and improve the precision of measurement. The triplicates will be performed at approximately 1-minute intervals. Each ECG recording (trace) will last 10 seconds. Repeat ECG will be performed until at least three 10-second ECG records per scheduled time-point meet the quality criteria

© ESTEVE (2010) & CONFIDENCIAL

set out in the SOM and the applicable SOP so to enable reading and analysing at least 5 complexes per derivation.

At screening, the four study periods and at follow-up, a total of 32 triplicate 12-lead ECG (96 single ECG) are scheduled to be recorded for each subject. This number will usually be exceeded when accounting for repeat measurements.

All recorded ECG will be reviewed by a Research Physician on an ongoing basis and the review be documented in the CRF. If a subject shows an abnormal ECG at any stage, additional safety recordings (including the use of 5 or 12-lead Holter equipment) may be made and the abnormality be followed to resolution if required.

12.4.2 Analysing and over-reading 12-lead ECG

Each electronic ECG will contain the ECG data as well as the result of the automated ECG analysis performed by the Marquette® 12SL™ ECG Analysis Program (MEAP), a program resident in each of the ECG machines.

All ECG and their associated automated interval measurements will subsequently be reviewed by qualified Cardiologists in accordance with the ICH E14 Guidance for Industry document and ICH E14 Implementation Working Group Questions and Answers document before any of the ECG are used for the thorough ECG analysis. The manual adjudication process applied in this study is also referred to in the ICH guidance and relevant literature as “manual over-read”, “computer-assisted” or “semi-automated” ECG measurements.

The following parameters on each ECG will be assessed by a cardiologist using the commercially available MUSE® in its latest version:

- QT interval
- RR interval
- Heart rate (HR)
- PR interval
- Presence or absence of U-wave
- Quantitative and qualitative ECG variations

Manual on-screen over-reading using electronic callipers in MUSE® will be performed by a small and select group of cardiologists with extensive experience with manual QT measurement (including on-screen measurement with electronic callipers). For all study ECG (Periods 1-4), the over-reading cardiologists will be blinded to time, date, treatment and any data identifying the subject. All ECG of a given subject will be over-read by the same cardiologist (or cardiologists in case manual adjustments of the automated measurement are necessary).

12.4.3 Recording of 24 hour 5-lead Holter ECG

Volunteers passing screening will undergo 24-hour ambulatory Holter assessments in order to assess their suitability for ECG measurements and in order to obtain ECG recordings allowing the assessment of their individual heart rate variability over a wide range of heart rates (Table 2). The devices will be fitted in accordance with RPL’s SOP. 24 hour Holter assessments will be obtained at screening (or within 3 months of dosing in Period 1).

© ESTEVE (2010) & CONFIDENCIAL

12.4.4 Extracting, Analysing and Over-Reading Holter ECG

Each Holter recording will be performed using Zymed DigiTrack Plus® Digital Holter Monitor, a compact Holter ambulatory ECG device that records and stores continuous ECGs. After the recording is finished and the device detached from the subject, the data will be downloaded and analysed using Philips 2010 Plus software. The Holter reports will be reviewed and signed off by a qualified cardiologist.

12.4.5 Telemetry

Telemetry will be obtained from 1 hour pre-dose on Day -1 and Day 1 until 24 hours post-dose in each treatment period. The telemetry monitoring may continue for longer at the discretion of the Investigator. Electrodes for telemetry will be positioned so as not to interfere with the electrodes for the 12-lead ECGs. Suitably trained staff will be responsible for monitoring of the telemetry.

12.5 Cognitive tests

Subjects will be asked to undergo computerised battery tests at the time points detailed in Table 2 and Table 3. These tests will include the following:

- Groton maze learning task (executive function)
- One card learning task (working memory and learning)
- Detection task (simple reaction time/psychomotor function)
- Identification task (choice reaction time)
- Sustained vigilance test (repeat of the detection task)
- VAS (polarities are included in Appendix 1).

All subjects will attend two training sessions on Day -2 of Period 1 only.

The detection (simple reaction time) and identification (choice reaction time) tests require the functional integrity of a number of cognitive processes in order to observe normal performance and also allow analysis of somnolence. Conducting both tests will allow the mean simple reaction time latency to be subtracted from the mean choice reaction time latency giving a measure of cognition which is independent of the motor components of the tasks.

Further details regarding the individual tests will be described in a separate SOM.

12.6 Pharmacokinetic Measurements

The timing of individual samples is described in the study plan (Table 2 and Table 3). Blood samples for determination of E-52862 concentrations in plasma will be collected up to 96 hours post-dose in study Periods 1 to 4. The date and time of collection and tube reference will be recorded on the appropriate CRF.

© ESTEVE (2010) & CONFIDENCIAL

12.6.1 Collection of pharmacokinetic samples

Venous blood samples collected into 4 mL tubes containing EDTA K2 as anticoagulant for the determination of concentrations of E-52862 and metabolites and placebo in plasma will be taken at the times presented in the study plan (Table 2 and Table 3).

For urine, the whole volume excreted on Period 1 from 0 to 12 h will be collected and measured for each subject. From this collection, an aliquot of 50 mL from each subject will be collected into tubes. Plasma and urine samples will be kept frozen at a nominal temperature of -80ºC until sent on dry ice to the bioanalytical laboratory for assay.

Further details for sample collection, storage and shipment will be detailed in a separate SOM. For blood volume see Section 12.7.

12.6.2 Determination of drug concentrations in pharmacokinetic samples

Plasma samples for determination of E-52862 and metabolite concentrations will be analysed by Departamento de Bioanálisis y ADME de Desarrollo using a validated LC/MS/MS method. Full details of the analytical methods used will be described in a separate bioanalytical report.

Plasma samples and urine samples may be used for metabolism purposes (including profiling, characterisation, isolation, and quantification) in order to gain further understanding of the pharmacokinetics of E-52862 in human. The findings of any such analyses will be reported separately.

Samples will be collected, stored and shipped as detailed in a separate SOM.

12.7 Volume of Blood Sampling

The total volume of blood that will be drawn from each subject in this study is as follows:

Table 5 Volume of Blood to be Drawn from Each Subject

Assessment		Sample Volume (mL)	Number of Samples	Total Volume (mL)
PK	E-52862 / placebo	4	64	256
Safety	Biochemistry	5	11	55
	Haematology	4	11	44
	Serology[a]	N/A	1	0
**Total**				355
Optional	Contingency[b]			195
**Maximum total**				550

[a] Serology can be tested from the clinical chemistry sample; therefore, no extra tube is required.

[b] Contingency for various repeats and to account for manufacturer change in sampling volumes

© ESTEVE (2010) & CONFIDENCIAL

13. ADVERSE EVENTS

The methods for collecting AEs are described below.

13.1 Adverse Events

13.1.1 Definitions

The definitions of AEs, adverse drug reactions (ADRs), SAEs and SUSARs are given below. It is of the utmost importance that all staff involved in the conduct of clinical research are familiar with the content of this section.

Adverse event (AE)

An AE is the development of an undesirable medical condition or the deterioration of a pre-existing medical condition following or during exposure to a pharmaceutical product, whether or not considered causally related to the product. An undesirable medical condition can be symptoms (e.g. nausea, chest pain), signs (e.g. tachycardia, enlarged liver), or the abnormal results of an investigation (e.g. laboratory findings, electrocardiogram). In clinical studies, an AE can include an undesirable medical condition occurring at any time, from the date informed consent was signed until the end of their participation in a study, i.e. the subject has discontinued or completed the study

The following factors should be considered when deciding if there is a “reasonable possibility” that an AE may have been caused by the drug.

- Time Course. Exposure to suspect drug. Has the subject actually received the suspect drug? Did the AE occur in a reasonable temporal relationship to the administration of the suspect drug?

- Consistency with known drug profile. Was the AE consistent with the previous knowledge of the suspect drug (pharmacology and toxicology) or drugs of the same pharmacological class? OR could the AE be anticipated from its pharmacological properties?

- Dechallenge experience. Did the AE resolve or improve on stopping or reducing the dose of the suspect drug?

- No alternative cause. The AE cannot be reasonably explained by another aetiology such as the underlying disease, other drugs, other host or environmental factors.

- Rechallenge experience. Did the AE reoccur if the suspected drug was reintroduced after having been stopped? Esteve would not normally recommend or support a rechallenge.

- Laboratory tests. A specific laboratory investigation (if performed) has confirmed the relationship?

A “reasonable possibility” could be considered to exist for an AE where one or more of these factors exist.

© ESTEVE (2010) & CONFIDENCIAL

In contrast, there would not be a “reasonable possibility” of causality if none of the above criteria apply or where there is evidence of exposure and a reasonable time course but any dechallenge (if performed) is negative or ambiguous or there is another more likely cause of the AE.

In difficult cases, other factors could be considered such as:

- Is this a recognised feature of overdose of the drug?
- Is there a known mechanism?

Ambiguous cases should be considered as being a “reasonable possibility” of a causal relationship unless further evidence becomes available to refute this.

Adverse Drug Reaction (ADR)

An ADR is any AE where a causal relationship with the IMP is at least a reasonable possibility.

Serious adverse event (SAE)

A SAE is an AE occurring during any study phase (i.e., from the date informed consent was signed until the end of their participation in a study), and at any dose of the investigational product, comparator or placebo, that fulfils one or more of the following criteria:

- Results in death
- Is immediately life-threatening
- Requires in-patient hospitalisation or prolongation of existing hospitalisation
- Results in persistent or significant disability or incapacity
- Is a congenital abnormality or birth defect
- Is an important medical event that may jeopardise the subject or may require medical intervention to prevent one of the outcomes listed above.

The causality of SAEs (i.e. their relationship to study treatment) will be assessed in the same way as for non-SAEs.

Note that SAEs that could be associated with any study procedure should also be reported.

Suspected Unexpected Serious Adverse Reactions (SUSAR)

A SUSAR is any serious AE where a causal relationship with the IMP is at least a reasonable possibility, but is not listed in the Investigator’s Brochure and/ or Summary of Product Characteristics.

© ESTEVE (2010) & CONFIDENCIAL

13.1.2 Recording of adverse events

AEs should be collected and recorded for each subject from the date informed consent was signed until the end of their participation in a study, i.e. the subject has discontinued or completed the study.

Following the end of the subject's participation in the study, the Principal Investigator (PI) or designated Physician should record and report AEs spontaneously reported by the subject if considered at least possibly related to study medication.

AEs may be volunteered spontaneously by the subject, or discovered by the study staff during physical examinations or by asking an open, non-leading question such as 'How have you been feeling since you were last asked?' All AEs and any required remedial action should be recorded in the subject's source documentation/ CRF page. The nature of the AE, date (and time, if known) of the AE onset, date (and time, if known) of AE resolution and severity of the AE will be documented together with the PI's or designated Physician's assessment of the seriousness of the AE and causal relationship to the study drug (at the time of assessment).

Findings and values related to physical examinations and measurements of ECG, BP, and pulse rate will be defined as AEs if they are considered clinically relevant deteriorations compared with baseline and pre-dose values, as judged by the Principal Investigator and/or the drug safety Physician.

Deterioration in laboratory values, BP, and pulse rate need not be reported as AEs. However, abnormal values that constitute an SAE must be reported and recorded as AEs.

13.1.3 Assessment of adverse events

13.1.3.1 AE Intensity

The following variables will be recorded for each AE: Onset, resolution, maximum intensity, action taken, outcome, causality, and whether it constitutes an SAE or not.

For grading the intensity of an AE or ADR, the following three categories must be used:

- mild (awareness of sign or symptom, but easily tolerated);
- moderate (discomfort sufficient to cause interference with normal activities);
- severe (incapacitating, with inability to perform normal activities).

It is important to distinguish between serious and severe AEs. Severity is a measure of intensity whereas seriousness is defined by the criteria in Section 13.1.1. An AE of severe intensity need not necessarily be considered serious. For example, nausea that persists for several hours may be considered severe nausea, but not a SAE. On the other hand, a stroke that results in only a limited degree of disability may be considered a mild stroke but would be an SAE.

In case of an overdose (accidental or deliberate), all symptoms associated with it should be reported as AEs.

© ESTEVE (2010) & CONFIDENCIAL

13.1.3.2 AE Causality

For each AE one of the following categories will be selected based on medical judgement, consideration for the definitions below and all contributing factors.

ADR related

Related

A clinical event, including a clinically significantly abnormal laboratory test or other measurement, occurs in a plausible time relationship to drug administration, and which concurrent disease or other drugs or chemicals cannot explain. The response to withdrawal of the drug (*dechallenge) should be clinically plausible. The event must be definitive pharmacologically or phenomenologically, using a satisfactory †rechallenge procedure if necessary.

Probably related

A clinical event, including a clinically significantly abnormal laboratory test or other measurement, with a reasonable time sequence to administration of the drug, unlikely to be attributed to concurrent disease or other drugs or chemicals, and which follows a clinically reasonable response on withdrawal (*dechallenge). †Rechallenge information is not required to fulfil this definition.

Possibly related

A clinical event, including a clinically significantly abnormal laboratory test or other measurement, with a reasonable time sequence to administration of the drug, but which could also be explained by concurrent disease or other drugs or chemicals. Information on drug withdrawal may be lacking or unclear.

Non-ADR related

Unrelated

A clinical event, including a clinically significantly abnormal laboratory test or other measurement, with little or no temporal relationship with drug administration, may have negative *dechallenge and †rechallenge information. Typically explained by extraneous factors (e.g. concomitant disease, environmental factors or other drugs or chemicals).

*Dechallenge is when a drug suspected of causing an AE is discontinued. If the symptoms of the AE disappear partially or completely, within a reasonable time from drug discontinuation, this is termed a positive dechallenge. If the symptoms continue despite withdrawal of the drug, this is termed a negative dechallenge. Note that there are exceptions when an AE does not disappear upon discontinuation of the drug, yet drug-relatedness clearly exists (for example, as in bone marrow suppression, fixed drug eruptions, or tardive dyskinesia).

†Rechallenge is when a drug suspected of causing an AE in a specific subject in the past is re-administered to that subject. If the AE recurs upon exposure, this is termed a positive rechallenge. If the AE does not recur, this is termed a negative rechallenge.

If the study is a double-blind study the causality assessment should be made under the assumption that the subject is receiving active study medication.

13.1.4 Reporting of SAEs

The procedures for reporting of SAEs will be agreed in detail between RPL and Esteve.

Any SAE / SUSAR will be notified by the Investigator to Esteve by email and fax.

Contact person: Dr. Neus Gascón

During business hours (from 9:00 to 17:00)

Pharmacovigilance department:

Tel.:+34 93 446 63 24

Tel.:+34 93 446 60 00 (Ext. 6120)

Fax:+34 93 446 62 63

e-mail: pharmacovigilance@esteve.es

Address: Avda. Mare de Déu de Montserrat, 221, 08041 - Barcelona, Spain

After business hours or on weekends:

Head of Pharmacovigilance department: Dr Neus Gascón Mobile: + 34 686 399 717

The time lines for notification are as follows:

	SUSARs (fatal and life threatening)	SUSARs (all others)	SAEs
RPL to Esteve	24 hours	24 hours	24 hours
RPL to REC	7 days	15 days	15 days
Esteve to Regulatory authority (MHRA)	7 days	15 days	Annual

The initial report will be followed up by a full written report within three working days unless no further information is available when the follow-up report will be provided as soon as possible when new information becomes available. Further follow-up reports will be provided as and when new information becomes available.

The event should be recorded on RPL's standard SAE form. SAEs/ SUSARs must be recorded and reported whether or not the Investigator considers the SAE/ SUSAR to be related to the IMP.

A summary of the outcome of the reaction and the Investigator's opinion of IMP relationship to the SAE/ SUSAR will accompany the SAE form, together with photocopies of the results and consultant reports, if and when available.

If any information relating to the study drug in a study becomes available after the submission of a final protocol to the competent authority which may impact on the conduct of the study, including but not limited to the risk and benefit evaluations underpinning approvals

© ESTEVE (2010) & CONFIDENCIAL

and volunteers consent, Esteve shall notify RPL in writing as soon as practically possible and the parties will agree, in writing, what steps need to be taken, if any.

14. QUALITY ASSURANCE AND QUALITY CONTROL

14.1 Quality Assurance (QA) and Quality Control (QC)

The sponsor has etchical, legal and scientific obligations to follow-up the study progress in accordance with clinical research principles and regulations.

To ensure Good Clinical Practice (GCP) compliance and compliance with all applicable regulatory requirements, Esteve or RPL may conduct a quality assurance audit.

The clinical study will be audited by the Quality Assurance Unit of Laboratorios del Dr. Esteve, S.A. according to Esteve audit plan. Therefore, direct access to source data and documentation will be provided for audit, for review by Ethics committee and for any regulatory inspection.

The clinical protocol, final report and the experimental phase (including RPL organisation, facilities and procedures) will be audited to assure the integrity of the data and to comply with the Good Clinical Practice regulation.

The audit will include but is not limited to: drug supplies tracking, presence of required study documents, informed consent process, medical records, general protocol compliance and comparison of data recorded in CRF against source documents.

A regulatory inspection of this study may be carried out by regulatory agencies. Such audits/inspections can occur at any time during or after completion of the study. If an audit or inspection occurs, the Investigator and institution agree to allow the auditor/inspector direct access to all relevant documents and to allocate his/her time and the time of his/her staff to the auditor/inspector to discuss findings and any relevant issues. The investigator is required to inform the Sponsor immediately of an inspection requested by a regulatory authority. The Sponsor will advise and help the Investigator in preparation for an inspection.

QC procedures at RPL will be implemented to ensure data recorded into the CRFs are accurate before CRFs are sent for data entry purposes. QC checks will be carried out on critical phases in the execution of the study. These control checks will be carried out according to the relevant SOPs. Records of these procedures will be documented and available for review.

14.2 Monitoring

During the study, a person designated by Esteve will have regular contact with the study site, including visits to:

- Provide information and support to the Investigators
- Confirm that facilities remain acceptable
- Confirm that the investigational team is adhering to the protocol, that data are being accurately and timely recorded in the CRFs, that biological samples are handled in

© ESTEVE (2010) & CONFIDENCIAL

accordance with the SOM and that investigational product accountability checks are being performed

- Perform source data verification (a comparison of the data in the CRFs with the subject's medical records at the hospital or practice, and other records relevant to the study) including verification of informed consent of participating subjects. This will require direct access to all original records for each subject (e.g., clinic charts)

- Ensure withdrawal of informed consent to the use of the subject's biological samples is reported and biological samples are identified and disposed of/destroyed accordingly, and the action is documented, and reported to the subject.

The CRAs designated by Esteve will be available between visits if the Investigators or other staff at the centre needs information and advice about the study conduct.

15. DATA MANAGEMENT

Data Management will be performed by the Data Management department of RPL. The data management process will be described in detail in the Data Handling Protocol (DHP).

The RPL Data Management department will be responsible for developing and maintaining the DHP; setting-up and validating the clinical study database; programming validation checks; entering data into the study database; reviewing data for accuracy, completeness and consistency between the CRF and the database; and verifying adherence to the clinical pharmacology study protocol and the DHP.

The study database will be constructed using Oracle Clinical version 4.5 based on the CRF data, using double independent data entry with second entry verification.

Laboratory data will be uploaded into the database as an electronic data transfer according to the validated transfer specification.

Data queries will be generated and resolved according to the DHP. All queries are documented individually on a Data Clarification Form (DCF) which is generated in Oracle Clinical. The DCF is a form designed to maintain an audit trail of modifications of the data in the clinical database and the justification for those modifications. All queries are resolved with the assistance of RPL clinical staff.

The database is locked after all queries are resolved and the database is declared clean. The database is then frozen following confirmation that the final error rate and QC checks are acceptable.

Standard SAS™ datasets are generated from the final study database ready for analyses. A complete audit trail of all corrections is available for inspection. All coding will be performed by RPL.

AEs, diagnoses from medical history and procedures from surgical history will be classified according to MedDRA (Medical Dictionary for Regulatory Activities), version 12.1 or higher.

Serious AEs in the clinical database will be reconciled with the safety database.

Final validated raw and derived SAS™ data sets will be transferred to Esteve according to the Data Transfer Specification, and following a format agreed by both sides.

15.1 Case Report Forms

CRFs will be used to record the data in the study. Data should be recorded legibly onto the CRFs in black ballpoint pen. Correction fluid or covering labels must not be used.

CRAs designated by Esteve will check data at the monitoring visits to the study site. The Investigator will ensure that the data in the CRFs are accurate, complete, and legible.

Data from the completed CRFs will be entered into RPL's clinical study database and validated under the direction of the Data Manager. Screening failures (subjects who signed consent to take part in the study but were not randomised) will not be entered into the clinical study database. Any missing, impossible (inconsistent with human life), or inconsistent recordings in the CRFs will be referred back to the Investigator using a DCF and be documented for each individual subject before clean file status is declared.

16. STATISTICAL EVALUATION AND CALCULATION

Statistical analyses will be performed using SAS™ version 9.2.

16.1 Pharmacokinetic Evaluation

16.1.1 Calculation or derivation of pharmacokinetic variables

The PK analyses will be performed using non-compartmental methods using SAS™ version 9.2 at RPL.

Calculations of PK parameters for E-52862 will be performed after a single oral dose of E-52862: C_{max}, t_{max}, $t_{½}$, AUC_{0-t}, $AUC_{0-\infty}$. Following is a description of each PK variable to be included in the analysis and the means by which each is calculated:

C_{max}	The observed maximum plasma concentration after single dose administration
t_{max}	The time to reach C_{max}
AUC_{0-t}	The area under the plasma concentration vs time curve from time zero to the last quantifiable concentration (C_{last}), calculated by the linear up-log down trapezoidal method.
$AUC_{0-\infty}$	The total area under the plasma concentration vs time curve, calculated by AUC_{0-t} + $AUC_{t-\infty}$, where $AUC_{t-\infty}$ is the residual area under the plasma concentration vs. time curve, extrapolated by C_{last}/λ_z (λ_z is the elimination rate constant estimated from individual linear regression of the terminal part of the log concentration vs time curve).
λ_z	the elimination rate constant estimated from individual linear regression of the terminal part of the log concentration vs time curve
$t_{½}$	The terminal elimination half-life, calculated by $0.693/\lambda_z$ (λ_z is the elimination rate constant estimated from individual linear regression of the terminal part of the log concentration vs time curve)

Actual sampling times will be used for all calculations of the PK parameters. If there is any doubt in the actual time a sample was taken, then the scheduled time will be used. Special consideration will be given to the estimation of λ_z and corresponding $t_{1/2}$ values. Values of λ_z will be calculated from a minimum of 3 data, if possible. These will be highlighted in the PK tables. Any values below the LLOQ of the assay before the t_{max} will be assumed to be zero. Values below the LLOQ which occur after the t_{max} will be ignored.

For the calculation of the pharmacokinetic parameters of the elimination half-life ($t_{1/2}$) associated with the terminal slope (λ_z) of a semi logarithmic concentration-time curve the following criteria will be applied:

1. The terminal slope of the semi logarithmic concentration-time curve will be in the elimination phase

2. The semi logarithmic concentration-time curve must include at a minimum 3 points.

3. The Rsq of the semi logarithmic concentration-time curve must be at a minimum 0.75

4. The percentage of extrapolated $AUC_{0-\infty}$ must be less than 25 %.

5. The $t_{1/2}$ must be ≤ (lambda z upper – lambda z lower), i.e., the elimination half-life must be equal or less than the time range used for its calculation.

If any of these criteria is not fulfilled then $t_{1/2}$ and $AUC_{0-\infty}$ will be NC (Not calculable).

Data values will be displayed to 2 decimal places. Values for t_{max} will be displayed in hours post start of dosing.

16.1.2 Analysis of pharmacokinetic variables

PK parameters will be analysed descriptively, AUC_{0-t}, $AUC_{0-\infty}$, C_{max}, $t_{½}$, and t_{max} will be summarised with arithmetic mean, geometric mean, minimum, median, maximum, SD, standard error, CVb(%), and 95% confidence limits of the means for each dose group. Log-transformed AUC_{0-t}, $AUC_{0-\infty}$, C_{max}, and $t_{½}$ values will be summarised with geometric mean, SD of the logs, 95% confidence limits, and CVb(%).

16.2 ECG Evaluation

The following parameters will be collected and reported:

- Uncorrected QT interval (ms)
- RR interval (ms)
- HR (bpm)
- Qualitative ECG variations to include:
    - morphological variations of the P wave and T wave,
    - occurrence of a U wave
    - occurrence of ventricular arrhythmia.
- Quantitative ECG variations including:
    - relative and absolute variations of P interval (ms),
    - relative and absolute variations PR interval (ms),
    - relative and absolute variations of QRS interval (ms).

© ESTEVE (2010) & CONFIDENCIAL

16.2.1 Calculation or derivation of ECG parameters

16.2.1.1 **Heart rate corrected QT interval corrected (QTc):**

Different QT corrections will be applied and the most accurate heart rate correction will be used to define the primary outcome of this study. This will be chosen after analysis of the blinded data.

- Individual correction (QTcI) (linear and log-log-linear models);
- Fridericia's correction (QTcF = QT/RR$^{0.33}$);
- Bazett's correction (QTcB= QT/RR$^{0.5}$).

16.2.1.2 **Baseline Correction (QTc^{baseline}):**

The baseline ECG recordings, at the beginning of each treatment period, are treatment and period specific and baseline ECG values are scheduled to match the "on-treatment" ECG sampling time points. All recordings are in triplicate and will be compliant with RPL's SOPs for the correct recording of ECG (in thorough QT/QTc studies). Mean and median QT/QTc values will be calculated for each time point (triplicate ECG) for subsequent analyses.

The primary baseline corrections will be calculated using averaged QTc baseline values (the mean of all median readings recorded for each time-point on the baseline Day -1). This single value (QTc^{baselineAV}) will be used to calculate ΔQTc for each study period.

The effect on QTc will be calculated as the placebo subtracted time matched difference as:

$\Delta\Delta QTc = (QTc^{active}-QTc^{baselineAV})–(QTc^{placebo}-QTc^{baselineAV})$ calculated for each of the 13 post dose ECG time points.

Holter ECG analysis will include quantitative and qualitative descriptions of the assessment periods and a comparison to all off treatment assessments.

16.2.2 Statistical methods for ECG analyses

Descriptive analyses will be performed to appropriately quantify and qualify and finding from the continuous ECG assessments. Summary statistics (n, arithmetic mean, median, minimum, and maximum) for all primary and secondary parameters will be calculated by treatment and time. Values outside the reference ranges and clinically significant values will be tabulated and flagged in the data listings.

16.2.2.1 Primary Analysis

The analysis of the interval changes will be based on the cross-over part of the study and will use the most appropriate heart rate correction (QTcI/QTcF/QTcB) and will be based on the change from average baseline. A linear mixed model with sequence, period, sex, treatment and time and time by treatment interaction as fixed effects, and baseline as covariate will be adapted, with subject as random effect. Two-sided 90% confidence intervals for the difference between each dose of E-52862 and placebo will be derived at each time point.

© ESTEVE (2010) & CONFIDENCIAL

Baseline will constitute the averaged baseline reference period used for the calculation of the baseline composite. The baselines are period specific in order to provide information on any possible carryover effects. Baseline ECG is scheduled to match the on-treatment clock time points for each treatment period.

16.2.2.2 Categorical Analysis

Categorical analyses will be performed to determine the number of subjects per treatment regimen and time who had an increase from baseline QTc greater than or equal to 30 msec and greater than or equal to 60 msec. Individual subjects who have a QTc value greater than or equal to 450 msec, greater than or equal to 480 msec and greater than or equal to 500msec will be summarised for each treatment regimen by gender.

16.2.2.3 Secondary Analysis

16.2.2.4 QT RR Relationship

Scatter plots of QT and QTc against RR will be produced for all data to visualize the best correction formula.

16.2.2.5 Concentration Effect Relationship

Plots of the differences with 90% CIs between both doses of E-52862 and placebo over time will be produced for all analyses to describe the concentration and effect relationship.

16.3 Cognitive test analysis

Cognitive tests will be summarised using descriptive statistics (arithmetic mean, geometric mean, minimum, median, maximum, SD). Cognitive test variables will be analysed by a mixed model with repeated measurements taking into account of the crossover design of this study. In the mixed model, Treatment (placebo, 500, 600 and 800 mg E-52862B), Time (0, 2 hours, 24 hours), Treatment x Time, Period (1, 2, 3 and 4), Sequence, and sex will be introduced as fixed effects, baseline measurement of a cognitive parameter as a covariate, and subject as a random effect. The treatment difference between active treatment group and placebo at each time point together with 95% confidence interval will be derived from the mixed model.

17. STATISTICAL METHODS AND SAMPLE SIZE DETERMINATION

17.1 Statistical Analysis Plan

A SAP will be written after finalising the protocol and before database lock. The specifications in this document will detail the implementation of all the planned statistical analyses in accordance with the principal features stated in the protocol.

17.2 Analysis Sets

17.2.1 General principles

The analysis of data will be based on different analysis sets according to the purpose of analysis, i.e., for safety and PK. Subject eligibility for each analysis set will be finalised before unblinding of the data. All data analyses will be performed using at least one of the following analysis sets.

The as-treated principle will be applied to all evaluations; i.e., subjects who received another treatment than the one assigned in the randomisation list will be analysed as belonging to the actual treatment group and not that assigned by randomisation. The placebo group will consist of all subjects treated with placebo regardless of treatment period.

17.2.2 Safety analysis set

All subjects who received at least one dose of randomised investigational product, E-52862 or placebo and for whom any post-dose data (including ECG and cognitive testing data) are available will be included in the safety analysis set.

17.2.3 Pharmacokinetic analysis set

The PK analysis set is defined as all subjects in the safety analysis set with available PK data. Subjects without protocol deviations affecting PK evaluations will be included in the PK analysis set. Subjects that receive that receive placebo will not be part of the PK analysis set.

17.3 Analysis of Other Safety Parameters

The objective of the evaluation of the safety parameters is to investigate any effects of the study drug on clinical safety and tolerability. All such parameters will be summarised by treatment. No formal hypothesis testing will be carried out.

17.3.1 Subject demographics

Continuous variables will be summarised using descriptive statistics (n, mean, SD, min, median, max) by treatment group. Categorical variables will be summarised in frequency tables (frequency and proportion) by treatment group.

17.3.2 Adverse events

AEs will be coded and reported according to MedDRA (Medical Dictionary for Regulatory Activities), version 12.1 or higher including system organ class and preferred term. If the severity or relationship to the IMP of an AE is missing, a worst-case scenario will be assumed (i.e. it will be set to severe or very likely/certain relationship). Separate summary tables will be included for treatment emergent AEs (i.e. commencing after dosing with the IMP) and non-treatment emergent AEs. Treatment emergent AEs will be assigned to the dose received preceding event onset. All AEs will be included in the data listings.

The number and percentage of subjects reporting AEs, serious AEs, IMP-related AEs, and AEs leading to withdrawal will be summarised.

© ESTEVE (2010) & CONFIDENCIAL

17.3.3 Laboratory parameters

Laboratory variables (haematology, biochemistry) will be summarised (by using mean, median, SD, minimum, maximum) at each time point including changes from baseline (i.e. from screening to follow-up). Values outside the reference ranges and clinically signififcant values will tabulated and flagged in the data listings. Descriptive graphs reflecting changes between baseline and on treatment values will be included. Urinalysis data will be listed only.

17.3.4 Vital signs

Vital signs data will be summarised by using mean, median, SD, minimum, and maximum at each time point. Any clinically significant values and changes will be tabulated and flagged in the data listings. Descriptive graphs reflecting changes between baseline and on treatment values will be included.

17.4 HANDLING OF MISSING AND INCOMPLETE DATA

17.4.1 Pharmacokinetic data

To derive the PK parameters, samples with plasma concentrations below the limit of quantification (LOQ) in early time-points will be treated as zero and plasma levels below the LOQ appearing in terminal samples will be omitted from the analysis.

17.4.2 ECG data

Missing QTc data will be treated as missing at random in the mixed model analysis. There will be no imputation of missing or incomplete data.

17.4.3 Cognitive test data

Missing cognitive test data will be treated as missing at random in the mixed model analysis. There will be no imputation of missing or incomplete data.

17.5 Sample size

Thirty two (32) healthy Caucasian males and females will be randomised for at least 28 subjects to complete all periods of the study. Of the randomised subjects at least 12 subjects (approximately 40%) should be male or female.

No formal sample size calculation has been performed; literature data (Zhang and Machado, 2008) suggests that thorough ECG studies with a sample size of 28 subjects and a SD of 8 ms are capable of detecting an 8 ms increase in QTc (α=0.05; β=0.9).

18. SPONSOR'S AND INVESTIGATOR'S RESPONSIBILITIES

This study will be conducted in accordance with current applicable regulations, ICH, European Union (EU) Directive 2001/20/EC, and local ethical and legal requirements.

© ESTEVE (2010) & CONFIDENCIAL

18.1 Sponsor's Responsibilities

18.1.1 GCP compliance

Esteve and any third party to whom aspects of the study management or monitoring have been delegated will undertake their roles for this study in compliance with all applicable regulations and ICH GCP Guidelines.

Visits to Investigator site will be conducted by representatives of Esteve to inspect study data, subjects' medical records, and CRFs in accordance with current GCP and the respective local and national government regulations and guidelines. Records and data may additionally be reviewed by auditors or by regulatory authorities.

18.1.2 Indemnity/liability and insurance

Esteve will adhere to the recommendations of the Association of British Pharmaceutical Industry (ABPI) Guidelines. A copy of the Indemnity document will be supplied to the Investigator before study initiation.

Esteve will ensure that suitable insurance cover is in place prior to the start of the study. An insurance certificate and as a minimum a listing of all exclusions will be supplied to RPL.

18.1.3 Protocol management

All protocols and amendments will be prepared by Esteve and/or RPL. If it becomes necessary to issue a protocol amendment during the course of the study Esteve will notify the Investigator and collect documented Investigator Agreement to the amendment.

18.1.4 End of trial notification

Esteve will submit an end of trial notification to the competent authority of the Member State within 90 days of the end of the trial in accordance with EU Directive 2001/20/EC. Esteve will forward copies of the end of trial notification documents to the Principal Investigator. The Principal Investigator will be responsible for submitting these to the Independent Ethics Committee (IEC) within 90 days of the end of the trial.

For the purposes of this notification, the end of the trial will be defined as the last subject/last end of study visit.

18.1.5 Submission of summary of clinical trial report to competent authorities of member states concerned and the Independent Ethics Committees

Esteve will provide a summary of the clinical trial report within one year of the end of the complete trial to the competent authority of the Member State concerned as required by the regulatory requirement and to comply with the Community guideline on Good Clinical Practice. Esteve will provide the Principal Investigator with a copy of the same summary to forward to the IEC.

18.2 Investigator's Responsibilities

18.2.1 GCP compliance

The Investigator must undertake to perform the study in accordance with ICH GCP Guidelines, EU Directive 2001/20/EC, and the applicable regulatory requirements.

It is the Investigator's responsibility to ensure that adequate time and appropriate resources are available at the study site prior to commitment to participate in this study. The Investigator should also be able to estimate or demonstrate a potential for recruiting the required number of suitable subjects within the agreed recruitment period.

The Investigator will maintain a list of appropriately qualified persons to whom the Investigator has delegated significant trial-related tasks. An up-to-date copy of the *curriculum vitae* for the Investigator, sub-investigator(s), and essential study staff will be provided to Esteve (or designee) before starting the study.

Agreement with the final Clinical Study Report will be documented by the signed and dated signature of the Principal Investigator, in compliance with Directive 75/318/EC, Directive 2001/83/EC, and ICH E3.

18.2.2 Regulatory approval

The Investigator (or a nominated designee) will ensure that Local Regulatory Authority requirements are met before the start of the study. The Investigator (or a nominated designee) will be responsible for the preparation, submission, and confirmation of receipt of any Regulatory Authority approvals required prior to release of investigational product prior to start of study.

18.2.3 Protocol adherence and Investigator agreement

The Investigator must adhere to the protocol as detailed in this document. The Investigator will be responsible for enrolling only those subjects who have met protocol eligibility criteria. The Investigators will be required to sign an Investigator Agreement to confirm acceptance and willingness to comply with the study protocol.

It is the Investigator's responsibility to ensure accurate and timely information is provided to the IEC at all phases during the study. Esteve will provide this information to the Principal Investigator who will be responsible for forwarding this to the IEC. In particular the appropriate approvals must be in place prior to recruitment, notification of any SAEs during the study must take place, and the IEC must be informed of study completion.

18.2.4 Documentation and retention of records

After completion of the study, all documents and data relating to the study will be kept in an orderly manner by the Investigator in a secure file and/or electronically. This file will be available for inspection by Esteve or their representatives. Essential documents must be retained for 2 years after the final marketing approval in an ICH region or at least 2 years have elapsed since the discontinuation of clinical development of the IMP. The Investigator must contact Esteve before destroying any study-related documentation and it is the

© ESTEVE (2010) & CONFIDENCIAL

responsibility of Esteve to inform the investigative site of when these documents can be destroyed. In addition, all subject records and other source documentation will be kept for a longer period if required by the applicable regulatory requirements.

18.3 Ethical Considerations

This protocol complies with the principles of the World Medical Assembly (Helsinki 1964) and subsequent amendments.

18.3.1 Informed consent

It is the responsibility of the Investigator to obtain written ICF from subjects. All consent documentation must be in accordance with applicable regulations, GCP and approved by an Ethics Committee.

The Principal Investigator will:

- Ensure that each subject is given full and adequate oral and written information about the nature, purpose, possible risk and benefit of the study.
- Ensure that each subject is notified that they are free to withdraw from the study at any time.
- Ensure that each subject is given the opportunity to ask questions and allowed time to consider the information provided.
- Ensure each subject provides signed and dated informed consent before conducting any procedure specifically for the study.
- Ensure the original, signed ICF(s) is/are stored in the Investigator's Study File.
- Ensure a copy of the signed ICF(s) is/are given to the subject.

The Principal Investigator will provide Esteve with a copy of the IEC approved consent forms, and a copy of the IEC written approval, prior to the start of the study. Additionally, if the IEC required modification of the sample ICF document provided by Esteve, the documentation supporting this requirement must be provided to Esteve.

18.3.2 Institutional Review Board or Independent Ethics Committee approval

It is the responsibility of the Investigator to submit this protocol, the informed consent document (approved by Esteve), relevant supporting information, and all types of subject recruitment information to the IEC for review, and all must be approved prior to study start. In addition, advertisements must be approved by the IEC prior to use at the site. Prior to implementing substantial changes in the study, Esteve and the IEC must also approve any revised informed consent documents and amendments to the protocol.

On the approval letter, the trial (title, protocol number, and version), the documents reviewed (protocol, informed consent material) and the date of review and actions taken should be clearly stated.

© ESTEVE (2010) & CONFIDENCIAL

Esteve is responsible for fulfilling all obligations (expedited reporting, e.g. SUSARs; periodic reporting, e.g. annual reports) regarding notification of Competent Authorities and Ethics Committees according to current international legislation. The relevant information related to the product for investigators is also the duty of Esteve.

18.4 Confidentiality

Data collected during this study may be used to support the development, registration, or marketing of medicinal product. Esteve will control all data collected during the study, and will abide by the EU Directive on Data Privacy concerning the processing and use of subjects' personal data. For the purpose of data privacy legislation, Esteve will be the data controller.

After subjects have consented to take part in the study, their medical records and the data collected during the study will be reviewed by Esteve and/or its representatives. These records and data may, in addition, be reviewed by the following: independent auditors who validate the data on behalf of Esteve; national or local regulatory authorities, and the IRB/IEC which gave its approval for this study to proceed.

Although subjects will be known by a unique number, their date of birth will also be collected and used to assist Esteve to verify the accuracy of the data, for example, that the results of study assessments are assigned to the correct subject. The results of this study containing the unique number, date of birth, and relevant medical information including ethnicity may be recorded and transferred to and used in other countries throughout the world, which may not afford the same level of protection that applies within the EU. The purpose of any such transfer would be to support regulatory submissions made by Esteve in such countries.

The Parties agree to comply with the relevant provisions of the Data Protection Act 2003 and any directions issued by the Information Commissioner in its processing of such Personal Data. All nominative information in the Research Subject's medical record will be kept in strict confidentiality. Nominative information shall mean the name, the address and all other personally identifiable information associated with a Research Subject's name. Esteve access to Research Subject's data shall be performed in such a way that no Research Subject could be identified by such data. According to that statement, data shall be considered as dissociated and, for that reason, requirements for personal data protection established on Spanish Law LO 15/1999 shall not be applied for Esteve.

If there are any contradictions in terms of confidentiality requirements, local law will prevail.

18.5 Publication Policy

RPL may not publish neither articles nor reports or make any presentations relating to the Study or referring to data, information or materials generated as part of the Study, in whole or in part, without the prior and express written consent of Esteve.

If RPL has interest in making publications and presentations relating to the Study in journals, at meetings or otherwise, such publications and presentations relating to the Study must be decided and determined by mutual agreement, and shall therefore permit such publications and presentations. Therefore, RPL shall provide to Esteve any proposed presentation or publication at least 180 (one hundred and eighty) working days prior to being disclosed and any other proposed publication at least 180 (one hundred and eighty) working days prior to being disclosed, and provided that Esteve shall have the right to require amendments to any

© ESTEVE (2010) & CONFIDENCIAL

such proposed presentation or publication on reasonable grounds including without limitation:

a) to ensure the accuracy of the presentation or publication;

b) to ensure that proprietary information is not inadvertently divulged;

c) to enable intellectual property rights to be secured;

d) to enable relevant supplementary information to be provided.

Authorship of any publications relating to the Study shall be determined by mutual agreement.

Esteve may require any proposed publication or presentation to be delayed for up to 6 (six) months to enable a patent application to be prepared and filed. The 6 (six) months period shall commence on the date of receipt of the proposed publication or presentation, or from the date when all relevant data from the Study are made available to Esteve, whichever is later.

© ESTEVE (2010) & CONFIDENCIAL

19. REFERENCES

Investigator Brochure, 2010
E-52862 Investigator's Brochure. Version number 4; May 2010

Cobos et al, 2008
Cobos EJ, Entrena JM, Nieto FR, Cendan Cm and Del Pozo E. Pharmacology and Therapeutic potential of Sigma1 Receptors Ligands. Curr Neuropharmacol 2008;6:344-366.

Guitart X et al, 2004
Guitart X, Codony X, Monroy X. Sigma receptors: biology and therapeutic potential. Psychopharmacology (Berl) 2004; 174:301-319.

Hayashi T et al, 2000
Hayashi T, Maurice T, Su TP. Ca(2+) signalling via sigma(1)-receptors: novel regulatory mechanism affecting intracellular Ca(2+) concentration. J Pharmacol Exp Ther 2000;293:788-798.

Hayashi T and Su TP, 2001
Hayashi T, Su TP. Regulating ankyrin dynamics: Roles of sigma-1 receptors. Proc Natl Acad Sci USA 2001;98:491-496.

National Cancer Institute Common Toxicity Criteria
http://evs.nci.nih.gov/ftp1/CTCAE/CTCAE_4.02_2009-09-15_QuickReference_5x7.pdf

ICH E14
ICH E14 The Clinical Evaluation of QT/QTc Interval Prolongation and Proarrhythmic Potential for Non-antiarrhythmic Drugs. 25-5-2005. International Conference on Harmonisation Step 4 Guideline, EMEA, CHMP/ICH/2/04.

Martina M et al, 2007
Martina M, Turcotte ME, Halman S, Bergeron R. The sigma-1 receptor modulates NMDA receptor synaptic transmission and plasticity via SK channels in rat hippocampus. J Physiol 2007;578:143-157.

Maurice T, 20004
Maurice T. Neurosteriods and sigma1 receptors, biochemical and behavioural relevance. Pharmacopsychiatry 2004;37:S171-182.

Monnet FP et al, 2006
Monnet FP, Maurice T. The sigma1 protein as a target for the non-genomic effects of neuro(active)steroids: molecular, physiological, and behavioural aspects. J Pharmacol Sci 2006;100:93-118.

Monnet FP et al, 1990
Monnet FP, Debonnel G, Junien JL, De Montigny C. N-methyly-D-aspartate-induced neuronal activation is selectively modulated by sigma receptors. Eur J Pharmacol 1990;179:441-445.

Su TP and Hayahi T, 2003
Su TP, Hayashi T. Understanding the molecular mechanism of sigma-1 receptors: towards a hypothesis that sigma-1 receptors are intracellular amplifiers for signal transduction. Curr Med Chem 2003;10:2073-2080.

Zhang and Machado, 2008
Zhang J and Machado S. Statistical issues including design and sample size calculation in thorough QT/QTc studies. J. Biopharm.Stat.18, 451-467, 2008.

20. APPENDICES

© ESTEVE (2010) & CONFIDENCIAL

APPENDIX 1 STANDARD VAS DIMENSIONS:

1)	Alert	Drowsy
2)	Calm	Excited
3)	Muzzy	Clear-headed
4)	Lethargic	Energetic
5)	Mentally slow	Quick-witted
6)	Tense	Relaxed
7)	Attentive	Dreamy
8)	Incompetent	Proficient
9)	Interested	Bored
10)	Depressed	Elated

© ESTEVE (2010) & CONFIDENCIAL
