# Supplementary material for: Single Doses up to 800 mg of E-52862 Do Not Prolong the QTc Interval – A Retrospective Validation by Pharmacokinetic-Pharmacodynamic Modelling of Electrocardiography Data Utilising the Effects of a Meal on QTc to Demonstrate ECG Assay Sensitivity
Source: PLoS One. 2015 Aug 20;10(8):e0136369. doi: 10.1371/journal.pone.0136369 (PMC4546378; doi:10.1371/journal.pone.0136369)
Supplement: S1 File — (DOCX) [file pone.0136369.s003.docx]

QTcF values at 6 h on Day -1.

| **QTcF** | **P1** | **P2** | **P3** | **P4** |
| --- | --- | --- | --- | --- |
| 1 | 418.699 | 421.8193 | 421.177 | 416.8619 |
| 2 | 417.4691 | 409.4196 | 406.8613 | 427.3376 |
| 3 | 455.2246 | 450.941 | 436.5274 | 435.9741 |
| 4 | 439.6697 | 399.8616 | 408.1243 | 411.2206 |
| 5 | 444.327 | 430.0431 | 423.8095 | 430.9349 |
| 6 | 389.8322 | 418.894 | 414.3386 | 421.0142 |
| 7 | 415.8458 | 412.7936 | 425.0845 | 420.3797 |
| 8 | 386.3454 | 412.912 | 403.624 | 396.0626 |
| 9 | 403.048 | 396.3657 | 379.4362 | 399.6562 |
| 10 | 395.7171 | 395.6056 | 400.7367 | 403.2203 |
| 11 | 402.461 | 404.1466 | 404.2866 | 404.466 |
| 12 | 415.9756 | 401.8177 | 404.5698 | 408.6701 |
| 13 | 408.2185 | 399.8434 | 404.9294 | 399.0975 |
| 14 | 362.9108 | 358.1939 | 360.7452 | 367.6269 |
| 15 | 384.9584 | 375.776 | 378.7232 | 375.4355 |
| 16 | 402.41 | 391.3482 | 399.1047 | 395.5387 |
| 17 | 417.7364 | 403.2417 | 402.5926 | 411.423 |
| 18 | 410.662 | 402.7929 | 401.7503 | 402.8409 |
| 19 | 411.3445 | 423.5476 | 426.2766 | 418.2806 |
| 20 | 391.6117 | 402.5465 | 410.6473 | 403.2019 |
| 21 | 407.3464 | 417.6124 | 412.1038 | 405.8689 |
| 22 | 399.621 | 399.5206 | 401.6922 | 408.7804 |
| 23 | 416.4579 | 408.8986 | 400.6719 | 419.8181 |
| 24 | 432.2291 | 423.8696 | 427.9631 | 423.4807 |
| 25 | 411.7914 | 410.9537 | 419.0383 | 418.2239 |
| 26 | 420.9455 |  |  |  |
| 27 | 392.2951 | 392.2901 | 409.7914 | 405.2412 |
| 28 | 398.4826 | 383.9516 | 391.4248 | 390.1164 |
| 29 | 409.628 | 402.3171 | 424.1151 | 410.2834 |
| 30 | 390.7109 | 394.0876 | 407.8132 | 398.4274 |
| 31 | 404.0079 | 397.921 | 399.2167 | 399.1383 |
| 32 | 394.4793 | 383.9311 | 403.8085 | 404.385 |

QTcF values at 8 h on Day -1.

| **QTcF** | **P1** | **P2** | **P3** | **P4** |
| --- | --- | --- | --- | --- |
| 1 | 404.6732 | 416.8404 | 410.3234 | 410.454 |
| 2 | 416.8663 | 410.4619 | 417.3299 | 420.0456 |
| 3 | 434.4258 | 440.916 | 435.723 | 428.5193 |
| 4 | 406.1614 | 397.45 | 407.2676 | 411.3493 |
| 5 | 428.4032 | 409.8959 | 421.5556 | 426.4742 |
| 6 | 394.0315 | 426.4942 | 417.1938 | 423.2069 |
| 7 | 414.6897 | 413.162 | 426.3992 | 420.3114 |
| 8 | 396.5729 | 392.8063 | 399.502 | 399.0396 |
| 9 | 398.3731 | 396.9743 | 380.1101 | 393.4949 |
| 10 | 396.0652 | 399.0355 | 396.0792 | 398.9268 |
| 11 | 408.5502 | 397.3506 | 409.5794 | 413.892 |
| 12 | 409.9879 | 404.2394 | 406.0233 | 403.3531 |
| 13 | 402.916 | 399.8395 | 401.8364 | 392.9915 |
| 14 | 367.3047 | 361.337 | 358.3254 | 364.9111 |
| 15 | 388.0752 | 375.7091 | 384.1152 | 376.253 |
| 16 | 392.6627 | 404.8833 | 402.0919 | 401.8294 |
| 17 | 409.7728 | 407.9458 | 412.2857 | 410.7948 |
| 18 | 406.294 | 400.843 | 408.1051 | 408.7383 |
| 19 | 403.407 | 414.7825 | 414.6279 | 414.2763 |
| 20 | 398.8466 | 396.7251 | 402.5961 | 415.5736 |
| 21 | 407.6251 | 414.4469 | 413.4164 | 413.3964 |
| 22 | 403.7709 | 399.339 | 406.8115 | 409.7365 |
| 23 | 410.828 | 423.4807 | 417.6372 | 412.3634 |
| 24 | 417.8528 | 424.3539 | 431.758 | 422.8293 |
| 25 | 419.1714 | 409.4919 | 420.7883 | 426.0369 |
| 26 | 419.7314 |  |  |  |
| 27 | 404.5978 | 402.0873 | 401.5929 | 410.4429 |
| 28 | 400.8352 | 384.1128 | 399.3545 | 398.1055 |
| 29 | 405.8839 | 401.6436 | 426.2392 | 420.6286 |
| 30 | 396.2586 | 396.9383 | 401.8361 | 399.3177 |
| 31 | 399.1511 | 383.936 | 395.6776 | 395.1348 |
| 32 | 395.5937 | 392.3504 | 402.9596 | 401.7971 |
